# Porcine placenta extract improves high-glucose-induced angiogenesis impairment

**DOI:** 10.1186/s12906-021-03243-z

**Published:** 2021-02-18

**Authors:** Chatchai Nensat, Worawat Songjang, Rutaiwan Tohtong, Tuangporn Suthiphongchai, Suchada Phimsen, Panthip Rattanasinganchan, Pornphimon Metheenukul, Sarawut Kumphune, Arunya Jiraviriyakul

**Affiliations:** 1 Integrative Biomedical Research Unit (IBRU), Faculty of Allied Health Sciences, Naresuan University, Phitsanulok, 65000 Thailand; 2 Graduate Program in Biomedical Sciences, Faculty of Allied Health Sciences, Naresuan University, Phitsanulok, 65000 Thailand; 3 Department of Medical Technology, Faculty of Allied Health Sciences, Naresuan University, Phitsanulok, 65000 Thailand; 4 Department of Biochemistry, Faculty of Science, Mahidol University, Bangkok, 10400 Thailand; 5 Department of Biochemistry, Faculty of Medical Science, Naresuan University, Phitsanulok, 65000 Thailand; 6 Faculty of Medical Technology, Huachiew Chalermprakiet University, Samut Prakan, 10540 Thailand; 7 Department of Veterinary Technology, Faculty of Veterinery Technology, Kasetsart University, Bangkok, 10900 Thailand; 8 Biomedical Engineering Institute (BMEI), Chiang Mai University, Chiang Mai, 50200 Thailand

**Keywords:** Porcine placenta extract, Angiogenesis impairment, Endothelial cell, Diabetes, Wound healing

## Abstract

**Background:**

High glucose (HG)-induced reactive oxygen species (ROS) overproduction impairs angiogenesis that is one pivotal factor of wound healing process. Angiogenesis impairment induces delayed wound healing, whereby it eventually leads to amputation in cases of poorly controlled diabetes with diabetic ulceration. Porcine placenta extract (PPE) is a natural waste product that comprises plenty of bioactive agents including growth factors and antioxidants. It was reported as an effective compound that prevents ROS generation. The goal of this study was to investigate the in vitro effect of PPE on HG-induced ROS-mediated angiogenesis impairment.

**Methods:**

Primary endothelial cells (HUVECs) and endothelial cell line (EA.hy926) were treated with HG in the presence of PPE. The endothelial cells (ECs) viability, intracellular ROS generation, migration, and angiogenesis were determined by MTT assay, DCFDA reagent, wound healing assay, and tube formation assay, respectively. Additionally, the molecular mechanism of PPE on HG-induced angiogenesis impairment was investigated by Western blot. The angiogenic growth factor secretion was also investigated by the sandwich ELISA technique.

**Results:**

HG in the presence of PPE significantly decreased intracellular ROS overproduction compared to HG alone. HG in the presence of PPE significantly increased ECs viability, migration, and angiogenesis compared to HG alone by showing recovery of PI3K/Akt/ERK1/2 activation. HG in the presence of PPE also decreased ECs apoptosis compared to HG alone by decreasing p53/Bax/cleaved caspase 9/cleaved caspase 3 levels and increasing Bcl 2 level.

**Conclusion:**

PPE attenuated HG-induced intracellular ROS overproduction that improved ECs viability, proliferation, migration, and angiogenesis by showing recovery of PI3K/Akt/ERK1/2 activation and inhibition of ECs apoptosis. This study suggests PPE ameliorated HG-induced ROS-mediated angiogenesis impairment, whereby it potentially provides an alternative treatment for diabetic wounds.

**Supplementary Information:**

The online version contains supplementary material available at 10.1186/s12906-021-03243-z.

## Background

Angiogenesis is a sophisticated and important process for wound healing. Since the formation of granulation tissue requires oxygen and nutrients to support dermal cells proliferation and migration [1, 2], the angiogenesis impairment leads to insufficient nutrients and oxygen for granulation forming, whereby it eventually mediates granulation tissue worsening and delayed wound healing [3]. Diabetes mellitus (DM) is a metabolic syndrome that causes angiogenesis imbalance [4]. The high glucose (HG)-induced overgrowth of angiogenesis initiates the proliferative diabetic retinopathy [5]. In contrast, the HG-induced angiogenesis impairment initiates delayed wound healing [6]. Even though there is a distinct physiological mechanism of HG-induced angiogenesis imbalance, the initial molecular mechanism acts in a similar manner [4]. HG generally induces cellular metabolic malfunction through the polyol pathway, advanced glycation end products (AGEs), the hexosamine pathway, and PKC fluctuation [7, 8]. Moreover, reactive oxygen species (ROS) also implicate as a cause of HG-induced cellular dysfunction through DNA disruption and oxidative stress activation [9]. Even though the paradox mechanism of HG-induced angiogenesis imbalance on retina and peripheral tissue is still unclear, the distinct location of endothelial cells and the imbalance of pro- and anti-angiogenic signals have been potentially implied as a different etiology of angiogenesis paradox [10]. However, the treatment of angiogenesis-induced proliferative diabetic retinopathy has been investigated by numerous researchers, whereby several novel procedures have instructed such as using anti-vascular endothelial growth factor (VEGF) agents, non-steroidal anti-inflammatory (NSAID) drugs, or laser therapy [11, 12]. Conversely, the treatment of angiogenesis impairment still challenges for improving delayed wound healing due to many related factors. Hence, the boarding bioactivities of integrated substances or compounds that potentially improves HG-induced angiogenesis impairment and delayed wound healing are still matters of interest.

The placenta is the temporary fetomaternal organ in the mammal uterus for providing nutrients and oxygen to the fetus, whereby it is expelled during the process of giving birth. The traditional medicine used the placenta as an antiaging therapy for decades because it comprises the plenty of growth factors, nucleic acids, and antioxidants. The placenta extract is currently derived from many creatures such as the porcine, bovine, and human with a supportive biological database, whereby the plenty of bioactive agents in placenta-derived extract cooperates and activates cellular bioactivities. For example, the porcine placenta extract (PPE) accelerates thermal-induced wound healing in rats because the PPE provokes basic fibroblast growth factor (bFGF) and transforming growth factor β1 (TGF-β1) expression in wound biopsy [13]. Moreover, the PPE improves dermatitis in rats by inhibiting ROS generation, which can prevent hyaluronic acids degradation and inflammatory responses, respectively [14]. Since PPE provides bioactivities in growth stimulation and ROS reduction, the treatment of PPE on HG-induced angiogenesis impairment that leads to delayed wound healing is then remarkable. Additionally, the PPE’s effect on angiogenesis remains unclear and lack of supportive information, especially in the HG model.

Therefore, the current study hypothesized that the treatment of PPE could improve HG-induced angiogenesis impairment by inhibiting ROS generation, preserving ECs viability and migration. To evaluate our hypothesis, we generated the HG model for ECs culture and treated ECs with various PPE concentrations.

## Methods

### Porcine placenta extract (PPE)

PPE solution was provided by Faculty of Sciences, Mahidol university, Bangkok, Thailand. In brief, the porcine placenta was cleaned and mechanically homogenized in phosphate buffer saline (PBS) solution. Then, the homogenate was sonicated and centrifuged at 4 °C for 1 h. Then, the supernatant was filtrated with 0.2 μm sterile filters.

### Chemicals and reagents

Vascular cell basal medium (ATCC PCS-100-030), endothelial cell growth kit-BBE (ATCC PCS-100-040), Penicillin-Streptomycin-Amphotericin B Solution (ATCC PCS-999-002), and Phenol red (ATCC PCS-999-001) were purchased from American Type Culture Collection (ATCC, Manassas, VA, USA). Dulbecco’s Modified Eagle Medium (DMEM), fetal bovine serum (FBS), and penicillin/streptomycin were purchased from Gibco (Gibco BRL, Life Technologies, Inc., NY, USA). D-glucose, Mannitol, polyvinylidenedifluoride (PVDF) membrane, and enhanced chemiluminescence (ECL) were purchased from Merck Millipore (Merck, Darmstadt, Germany). Primary antibodies against phosphorylated-PI3K, phosphorylated-Akt, total-Akt, phosphorylated-ERK1/2, total-ERK1/2, p53, caspase 3, caspase 9, Bax, Bcl 2, and β-Actin were purchased from Cell Signaling Technology (Cell Signaling Technology, Inc., Danvers, MA, USA). Other chemicals and reagents were purchased from Sigma Aldrich (Sigma, St. Louis, MO, USA).

### Cell culture

The human umbilical vein cell lines (EA.hy926) (ATCC-CRL2922) and the primary umbilical vein endothelial cells (HUVECs) (ATCC PCS-100-013) were purchased from American Type Culture Collection (ATCC, Manassas, VA, USA). EA.hy926 cells were cultured in DMEM supplemented with 10% FBS and 1% penicillin/streptomycin. HUVECs were cultured in vascular basal medium supplemented with endothelial cell growth kit-BBE, and penicillin-streptomycin-amphotericin B solution. Cells were maintained in humidified atmosphere of 95% air and 5% carbon dioxide at 37 °C until 80% confluence. HUVECs of passage 6–10 and EA.hy926 cells of passage 6–15 were used in all experiments.

### Modeling of HG condition and optimization of PPE concentration

EA.hy926 cells were seeded in 96-well plate (5 × 10^3^ cells/well) and incubated overnight. The intracellular ROS generation and the ECs viability were determined to optimize glucose concentration as a HG model. The ECs viability was determined to optimize PPE concentration as efficient concentrations.

### Intracellular ROS generation

The determination of intracellular ROS generation was performed by using DCFDA/H2DCFDA- cellular ROS assay kit (Abcam, Cambridge, UK). The principle of this assay is 2′,7′-Dichlorodi-hydrofluorescein diacetate (DCFDA) deacetylated to nonfluorescent compound by membrane esterase, whereby the oxidation of nonfluorescent compound with intracellular ROS constitutes fluorescent compound (DCF) in cells. The fluorescence intensity is proportional to intracellular ROS quantity [15]. HUVECs and EA.hy926 cells were seeded in clear bottom black 96-well plate (1 × 10^4^ cells/well) and incubated overnight. Then, cells were washed and treated with normal glucose (NG) alone, high glucose (HG) alone, and HG in the presence of various PPE concentrations. NG in the presence of hydrogen peroxide (H_2_O_2_) 100 μM is a positive control of HG alone. HG in the presence of N-acetylcysteine (NAC) 15 μg/mL is a positive control of HG in the presence of PPE. After treatment for 12 h, cells were washed with 1X dilution buffer and incubated with DCFDA 5 μM in dark at 37 °C for 30 mins. The fluorescence was immediately detected by using fluorescence microscopy with FITC filter and quantified by using microplate reader at 485 nm excitation/535 nm emission.

### Cell viability by MTT assay

The determination of cell viability was performed by using MTT (3-[4,5-dimethylthiazol-2-yl]-2,5-diphenyltetrazolium bromide) assay [15]. In brief, ECs were seeded in 96-well plate (5 × 10^3^ cells/well for EA.hy926 cells and 8 × 10^3^ cells/well for HUVECs) and incubated overnight. Then, ECs were washed and treated with NG alone, HG alone, and HG in the presence of various PPE concentrations. After treatment, the medium was discarded and incubated with MTT 0.5 mg/mL in serum-free medium at 37 °C for 4 h. The formazan crystal was solubilized by adding DMSO. The optical density (OD) was determined by using microplate reader (PerkinElmer, MA, USA) at 490 nm.

### Cell migration by wound healing assay

HUVECs were seeded in 24-well plate and cultured until greater than 70% confluence. Then, the wounding was generated by using a p200 pipette tip. Cell debris and dislodged cells were removed by washing twice with PBS. After treatment, ECs migration was observed and photographed at 0, 6, 12, and 24 h by using inverted microscopy. The wound reduction was measured and calculated as a following equation by using ImageJ: [1 – (A_specific timepoint_/A_t = 0_)] × 100%, A_specific timepoint_ represents a wound area at specific timepoint (6, 12, 24 h) and A_t = 0_ represents a wound area at initial timepoint (0 h).

### Endothelial tube formation

The tube formation assay was performed according to CORNING® protocol. In brief, the precooled 24-well plate was coated with chilled Corning® Matrigel® Matrix 10 mg/mL for 250 μL in each well and solidified at 37 °C for 30 mins in incubator. Then, each mixture of treatment medium with HUVECs was prepared for 300 μL, whereby each mixture contains 1.2 × 10^5^ cells of HUVECs. After that, the gel-coated plate was overlaid with each mixture in each well and incubated at 37 °C. The tube formation was observed every 2 h. The tubule quantity was measured by photographing with inverted microscopy and calculated by using angiogenesis plugin in ImageJ.

### Western blot

After treatment according to indicated protocol, the intracellular protein was harvested by using NP-40 lysis buffer contained with protease inhibitor cocktail (AMRESCO, OH, USA). Then, each lysate was placed on ice and mixed every 15 mins for 1 h. Then, each lysate was centrifuged at 1.2 × 10^4^ rpm for 10 mins. The supernatant was collected and measured the protein concentration by using Bradford assay. Subsequently, the protein was separated by sodium dodecyl sulfate polyacrylamide gel electrophoresis and transferred protein bands to the PVDF membrane. The non-specific binding was blocked with 5% skim milk buffer for 1 h and washed twice with 1X TBST buffer. The membrane was probed with primary antibody overnight and incubated with horseradish peroxide-linked anti-rabbit antibody (Cell Signaling Technology, Inc., Danvers, MA, USA). The image was developed by adding ECL reagent and placing in Chemidoc™ XRS (Bio-rad, CA, USA).

### Angiogenic growth factor secretion by using the sandwich ELISA technique

Basic fibroblast growth factor (bFGF) and epidermal growth factor (EGF) were determined by using human FGF-basic standard ABTS ELISA development kit and human EGF standard ABTS ELISA development kit (PeproTech Inc., Rocky Hill, NJ, USA), respectively. After treatment according to indicated protocol, the supernatant was collected and discarded cell debris by centrifugation. Then, the supernatant was incubated in pre-blocked capture antibody-coated 96-well plate for 2 h at room temperature (RT). Then, the detection antibody was added and incubated for 2 h at RT. After that, the avidin-HRP-conjugated anti IgG was added and incubated for 30 mins at RT. After adding a substrate, the luminescence was determined by using microplate reader at 450/605 nm.

### Statistical analysis

All data is shown as mean ± SEM and analyzed the significant difference by using ANOVA with appropriate post-hoc comparison analysis. A *p*-value < 0.05 was considered as statistically significant difference. The statistical analysis was performed by using commercially available software (GraphPad Prism version 9, San Diego, CA, USA).

## Results

### Modeling of HG and optimization of PPE concentrations

**Fig. 1:**
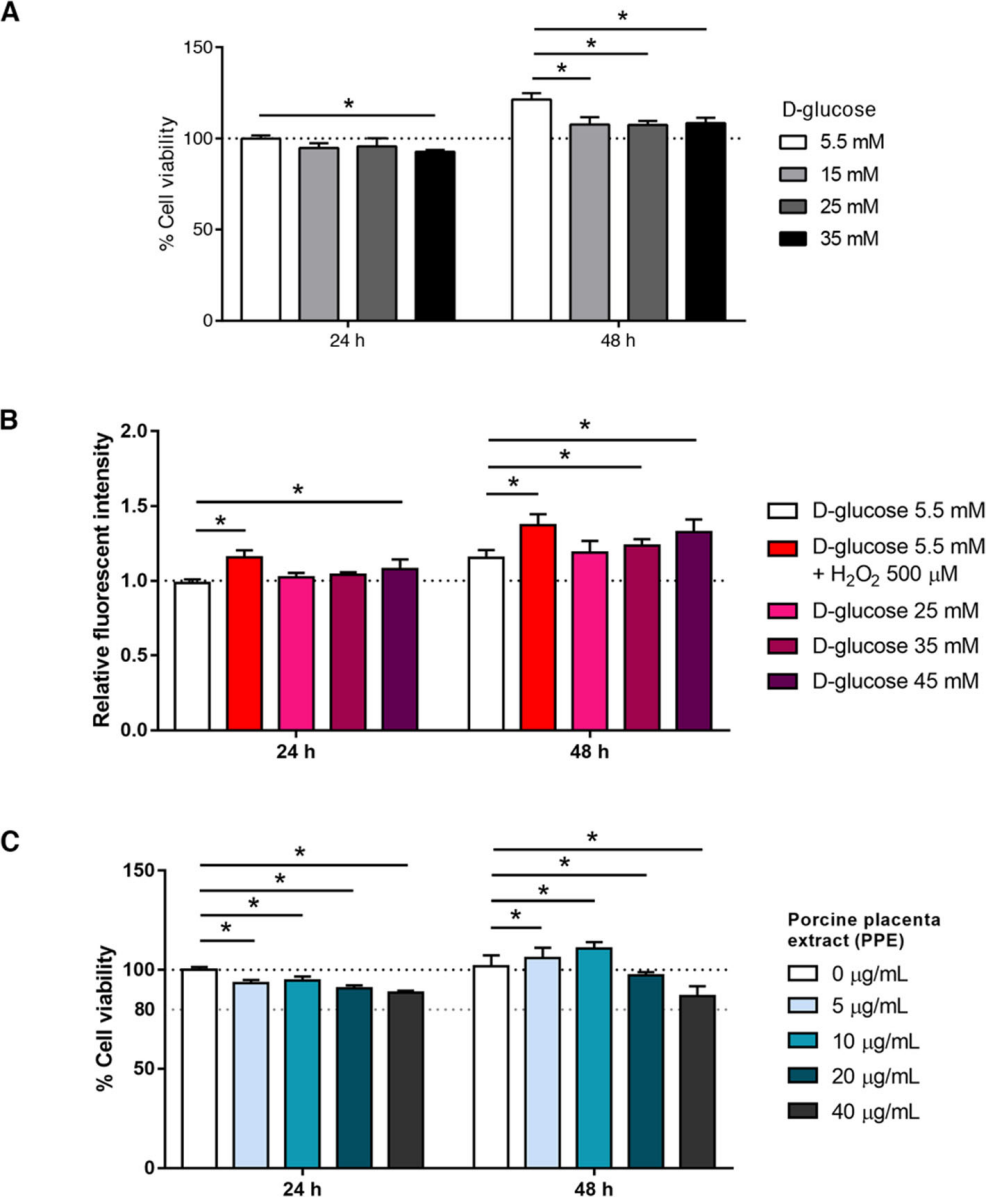
Modeling of HG and optimization of PPE concentrations. **a** EA.hy926 cells were treated with various glucose concentrations and determined ECs viability by using MTT assay at 24 and 48 h. Glucose 5.5 mM represents normal glucose as a control **b** EA.hy926 cells were treated with various glucose concentrations and quantified the intracellular ROS generation by using DCFDA reagent at 24 and 48 h. H_2_O_2_ 500 μM in normal glucose represents a positive control. **c** Ea.hy926 cells were treated with various PPE concentrations and determined ECs viability. PPE 0 μg/mL represents a control. Each bar represents in mean ± SEM. * *p* < 0.05 (One way-ANOVA, 3-time independent, *n* = 9)

EA.hy926 cells were treated with various glucose concentrations at 5.5, 15, 25, and 35 mM and incubated for 24 and 48 h. Glucose 5.5 mM represents NG and glucose 15, 25, 35 represent HG. The results indicated that glucose 35 mM significantly reduced ECs viability at 24 and 48 h compared to NG. Glucose 15 and 25 mM did not show any difference compared to NG at 24 h. However, there is a significant difference of glucose 15 and 25 mM compared to NG at 48 h (Fig. 1a). EA.hy926 cells were also treated with various glucose concentrations and determined intracellular ROS generation. The results indicated that glucose greater than or equal to 35 mM significantly increased intracellular ROS generation compared to NG (Fig. 1b). Therefore, glucose 35 mM was defined to the HG model in further experiments due to its minimal concentration showed the statistically significant difference on ECs viability and intracellular ROS generation.

The PPE concentration was optimized by determining ECs viability. EA.hy926 cells were treated with various PPE concentrations at 0, 5, 10, 20, and 40 μg/mL for 24 and 48 h. The results indicated that PPE significantly increased ECs viability compared to a control, especially PPE 5 and 10 μg/mL at 48 h. Even though PPE did not show ECs viable enhancement at 24 h, PPE did not decrease below 80% of ECs viability (Fig. 1c). Therefore, PPE 5 and 10 μg/mL were applied in HG model and investigated in further experiments.

### PPE improved HG-induced endothelial cell viable impairment and reduced HG-induced intracellular ROS generation

**Fig. 2:**
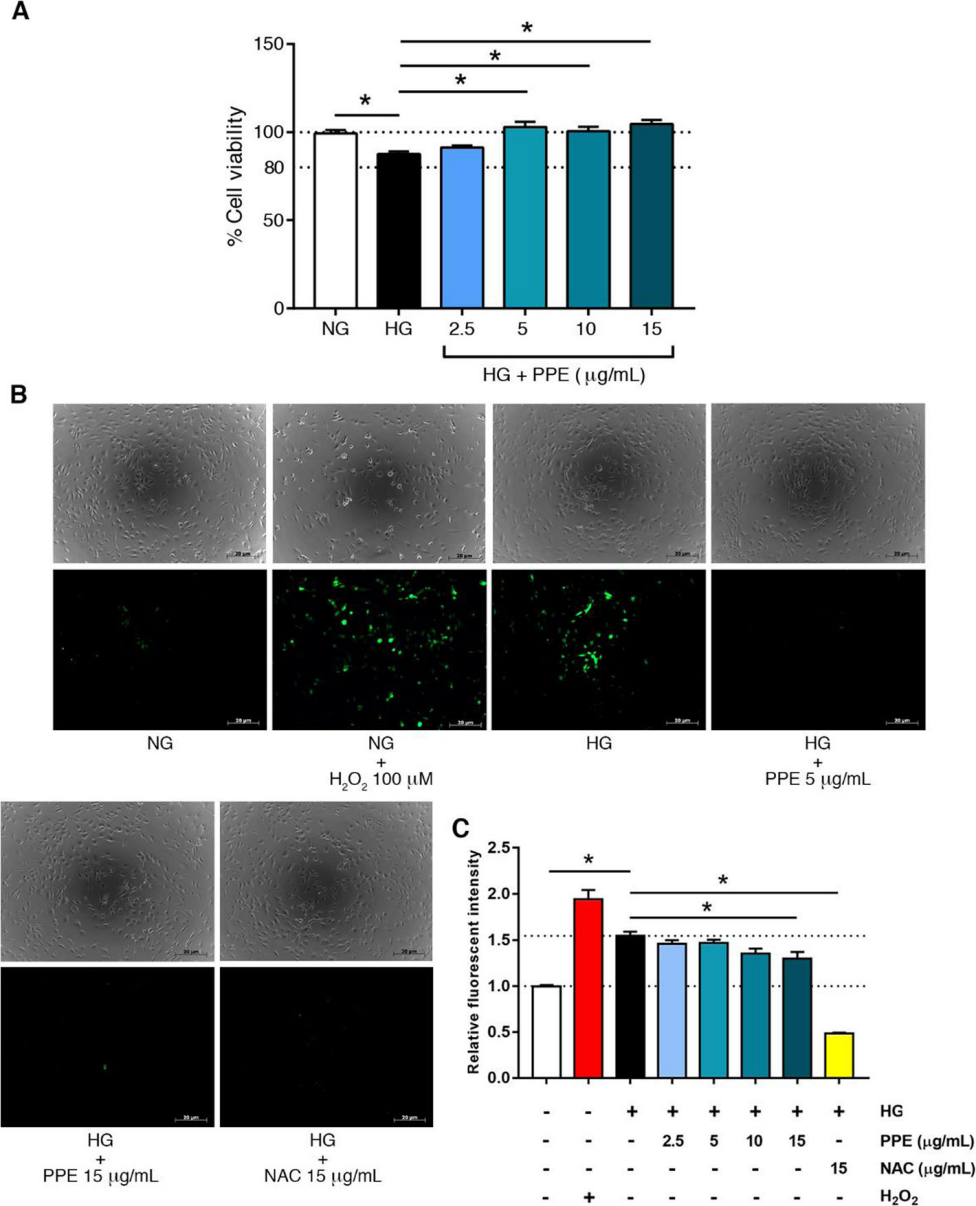
PPE improved HG-induced ECs viable impairment and reduced HG-induced intracellular ROS generation. **a** HUVECs were treated with optimal PPE concentrations and determined ECs viability by using MTT assay. **b, c** HUVECs and EA.hy926 cells were treated with optimal PPE concentrations and quantified the intracellular ROS generation by using DCFDA reagent. NAC 15 μg/mL represents a positive control of PPE treatment. **b** The fluorescence was observed under fluorescence microscopy with FITC filter. **c** The fluorescence was also determined by microplate reader at 485 nm excitation/535 nm emission. Each bar represents in mean ± SEM. * *p* < 0.05 (One way-ANOVA, 2-time independent, *n* = 6)

HUVECs and EA.hy926 cells were treated with NG alone, HG alone, and HG in the presence of various PPE concentrations at 2.5, 5, 10, and 15 μg/mL. The results demonstrated that HG in the presence of PPE 5, 10, 15 μg/mL significantly increased ECs viability compared to HG alone. HG alone also expressed the ECs viable reduction compared to the NG alone with significant difference (Fig. 2a).

HG in the presence of PPE expressed the intracellular ROS reduction. HUVECs and EA.hy926 cells were treated according to the indicated protocol. The results indicated that HG alone significantly increased intracellular ROS generation compared to NG alone. Interestingly, HG in the presence of PPE 15 μg/mL significantly decreased intracellular ROS generation compared to HG alone. NAC is a ROS scavenger that was utilized as a positive control of PPE treatment. HG in the presence of NAC 15 μg/mL also significantly decreased intracellular ROS generation compared to HG alone (Fig. 2b and Fig. 2c).

### PPE improved HG-induced endothelial cell migrative impairment

**Fig. 3:**
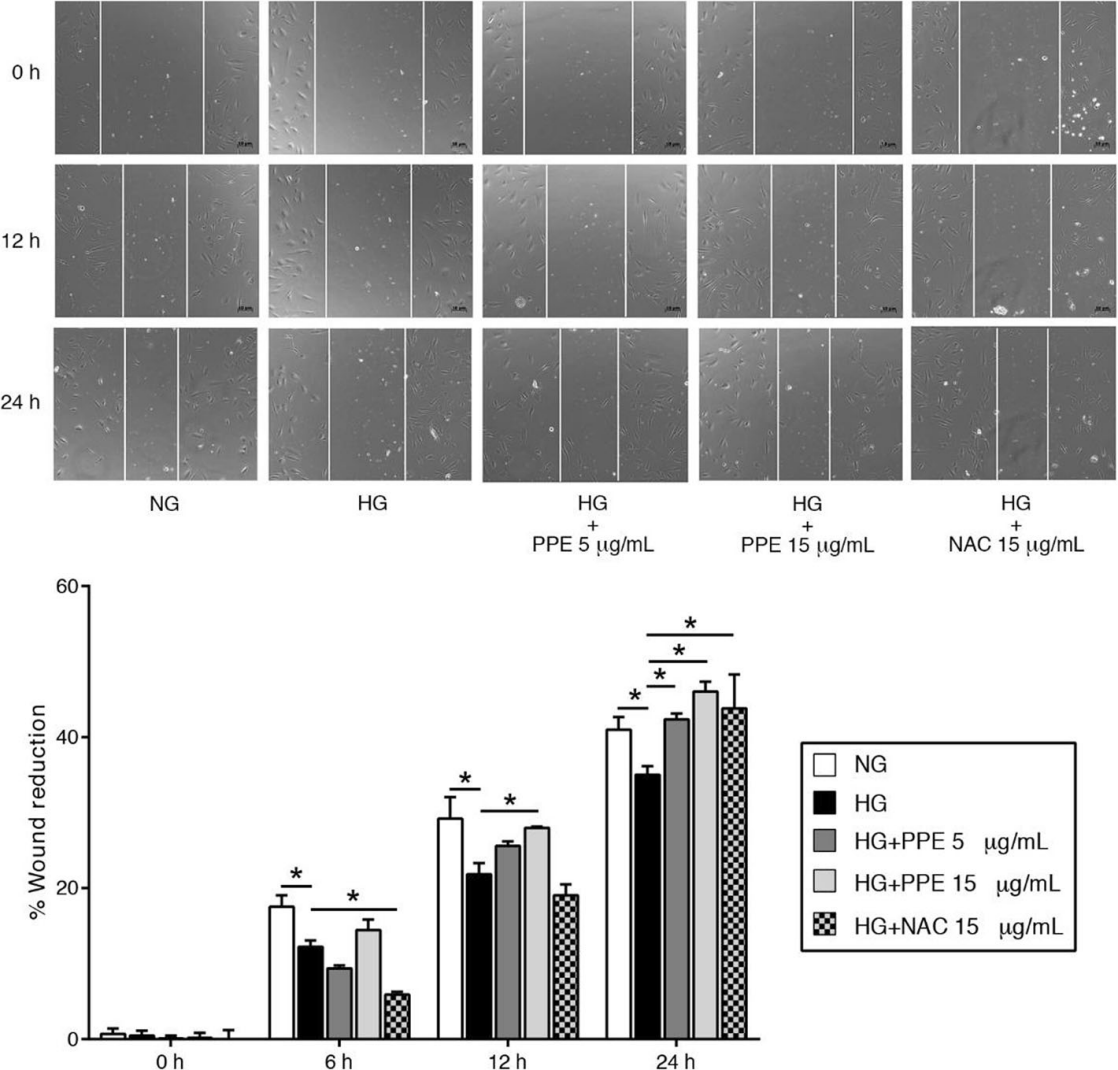
PPE improved HG-induced ECs migrative impairment. HUVECs were treated with optimal PPE concentrations after wounding. The wound reduction was observed at 0, 6, 12, 24 h. The percentage of wound reduction was calculated according to indicated equation. Each bar represents in mean ± SEM. * *p* < 0.05 (One way-ANOVA, 2-time independent, *n* = 6)

HUVECs were treated with NG alone, HG alone, and HG in the presence of PPE 5 and 15 μg/mL. The results demonstrated that HG alone significantly reduced the percentage of wound reduction compared to NG alone at every timepoint. Interestingly, HG in the presence of PPE accelerated wound reduction compared to HG alone. Especially, HG in the presence of PPE 15 μg/mL significantly increased the percentage of wound reduction compared to HG alone at every timepoint. HG in the presence of NAC 15 μg/mL also increased the percentage of wound reduction compared to HG alone at 24 h (Fig. 3).

### PPE improved HG-induced angiogenesis impairment

**Fig. 4:**
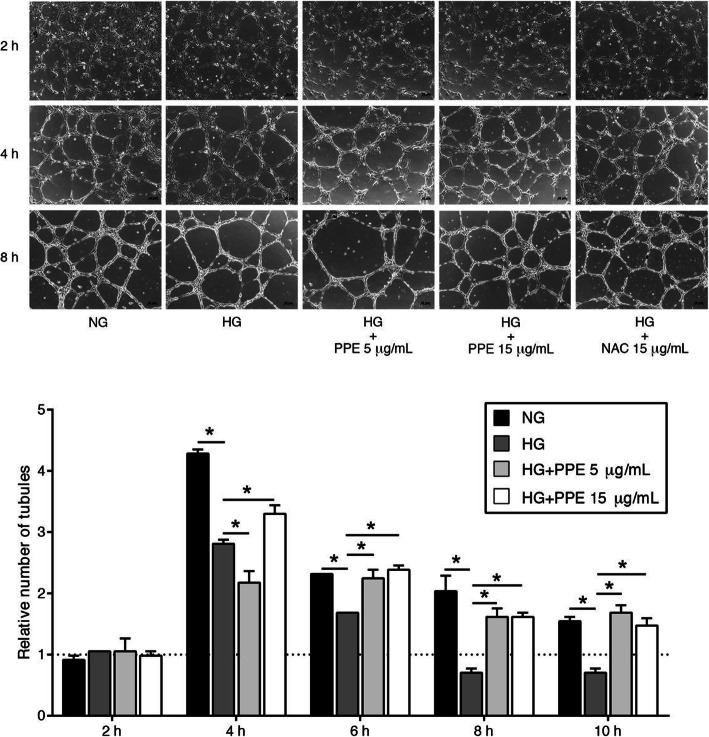
PPE improved HG-induced angiogenesis impairment. HUVECs were treated with optimal PPE concentrations. The tube formation was observed every 2 h under inverted microscopy. Each bar represents in mean ± SEM. * *p* < 0.05 (One way-ANOVA, 2-time independent, *n* = 6)

HUVECs were treated with NG alone, HG alone, and HG in the presence of PPE 5 and 15 μg/mL and the tube formation was observed every 2 h. The results demonstrated that HG alone significantly reduced the number of tubules compared to NG alone. Interestingly, HG in the presence of PPE 5 and 15 μg/mL significantly increased the number of tubules compared to HG alone (Fig. 4). HG in the presence of NAC 15 μg/mL also expressed the enhancement of tubules compared to HG alone.

### PPE improved HG-reduced angiogenic growth factor secretion

**Fig. 5:**
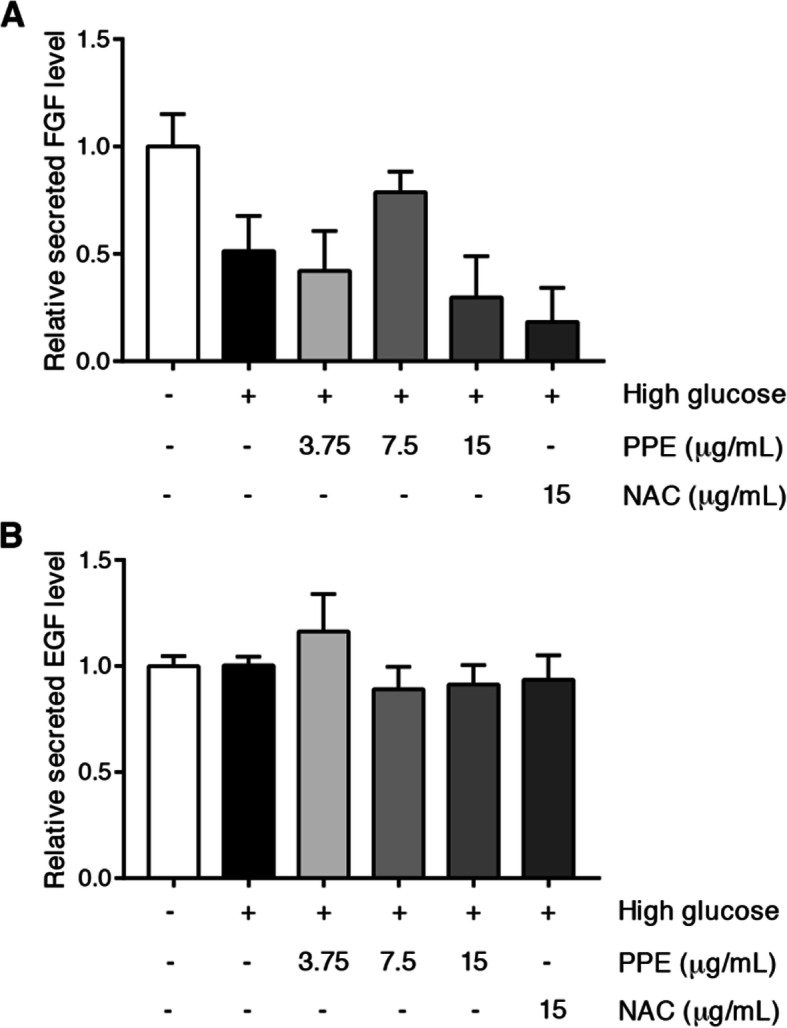
PPE improved HG-reduced angiogenic growth factor secretion. HUVECs were treated according to indicated protocol. **a** The bFGF in supernatant was detected by using human FGF-basic standard ABTS ELISA development kit. **b** The EGF in supernatant was detected by using human EGF standard ABTS ELISA development kit. Each bar represents in mean ± SEM. * *p* < 0.05 (One way-ANOVA, 2-time independent, *n* = 6)

HUVECs were treated with NG alone, HG alone, and HG in the presence of PPE 3.75, 7.5, and 15 μg/mL for 48 h. Subsequently, the supernatant was collected and determined bFGF and EGF secretions. The results demonstrated that HG alone reduced bFGF secretion compared to NG alone. Interestingly, HG in the presence of PPE increased bFGF secretion compared to HG alone (Fig. 5a). However, HG alone did not show the declination of EGF secretion compared to NG alone (Fig. 5b). The VEGF was also determined but it could not be detected in the supernatant.

### PPE improved HG-reduced PI3K/Akt/ERK1/2 activation

**Fig. 6:**
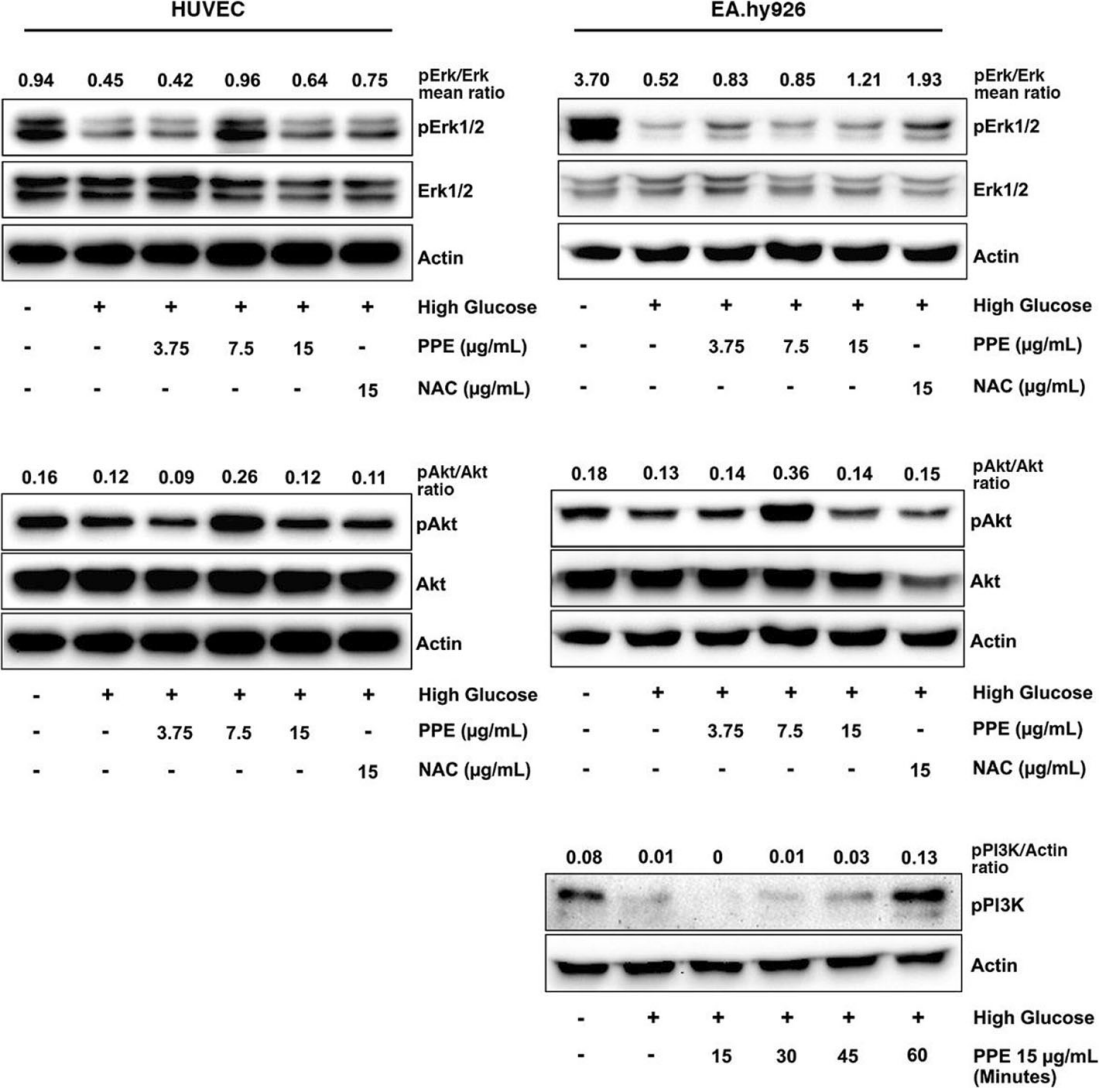
PPE improved HG-reduced PI3K/Akt/ERK1/2 activation. ECs were treated, harvested, and investigated the pro-survival signaling pathway by using Western Blot. The ratio of phospho- protein intensity and total-protein intensity was represented in individual lane. The ratio of phospho-PI3K intensity and β-actin intensity was represented in individual lane

To determine the regulatory effect of PPE on PI3K/Akt/ERK1/2 phosphorylation in HG, ECs were pretreated with NG and HG for 3 days. Subsequently, ECs were treated with indicated protocol in individual NG- or HG-pretreated group. ECs were harvested and lysed with lysis buffer. The results illustrated that HG alone reduced the phosphorylation of ERK1/2, Akt, and PI3K compared to NG alone. In contrast, HG in the presence of PPE increased the phosphorylation of ERK1/2, Akt, and PI3K compared to HG alone. Concomitantly, HG in the presence of NAC also increased the phosphorylation of ERK1/2, Akt compared to HG alone (Fig. 6).

### PPE attenuated HG-induced endothelial cell apoptosis

**Fig. 7:**
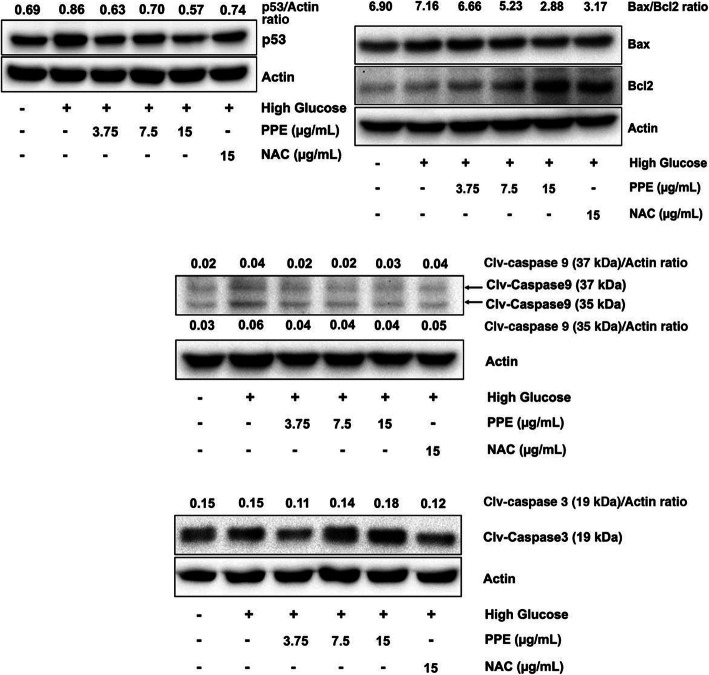
PPE attenuated HG-induced apoptotic regulatory molecules. ECs were treated, harvested, and investigated p53, Bax, Bcl 2, cleaved caspase 9, and cleaved caspase 3 by using Western Blot. The ratio of apoptotic regulatory molecule intensity and β-actin intensity was represented in individual lane

To determine the regulatory effect of PPE on p53/Bax/Cleaved caspase 9/Cleaved caspase 3 level in HG, ECs were treated with NG alone, HG alone, and HG in the presence of various PPE concentrations. Subsequently, ECs were harvested and lysed with NP-40 lysis buffer. The results illustrated that HG alone increased p53, Bax, cleaved caspase 9, and cleaved caspase 3 levels compared to NG alone. In contrast, HG in the presence of PPE reduced p53, Bax, cleaved caspase 9, and cleaved caspase 3 levels compared to HG alone. HG in the presence of NAC also reduced p53, Bax, cleaved caspase 9, and cleaved caspase 3 levels compared to HG alone. Concomitantly, HG in the presence of PPE also increased Bcl 2 level compared to HG alone. The Bax/Bcl 2 ratio of NG alone, HG alone, HG in the presence of PPE and NAC are 6.90, 7.16, 6,66, 5.23, 2.88, and 3.17, respectively (Fig. 7).

## Discussion

**Fig. 8:**
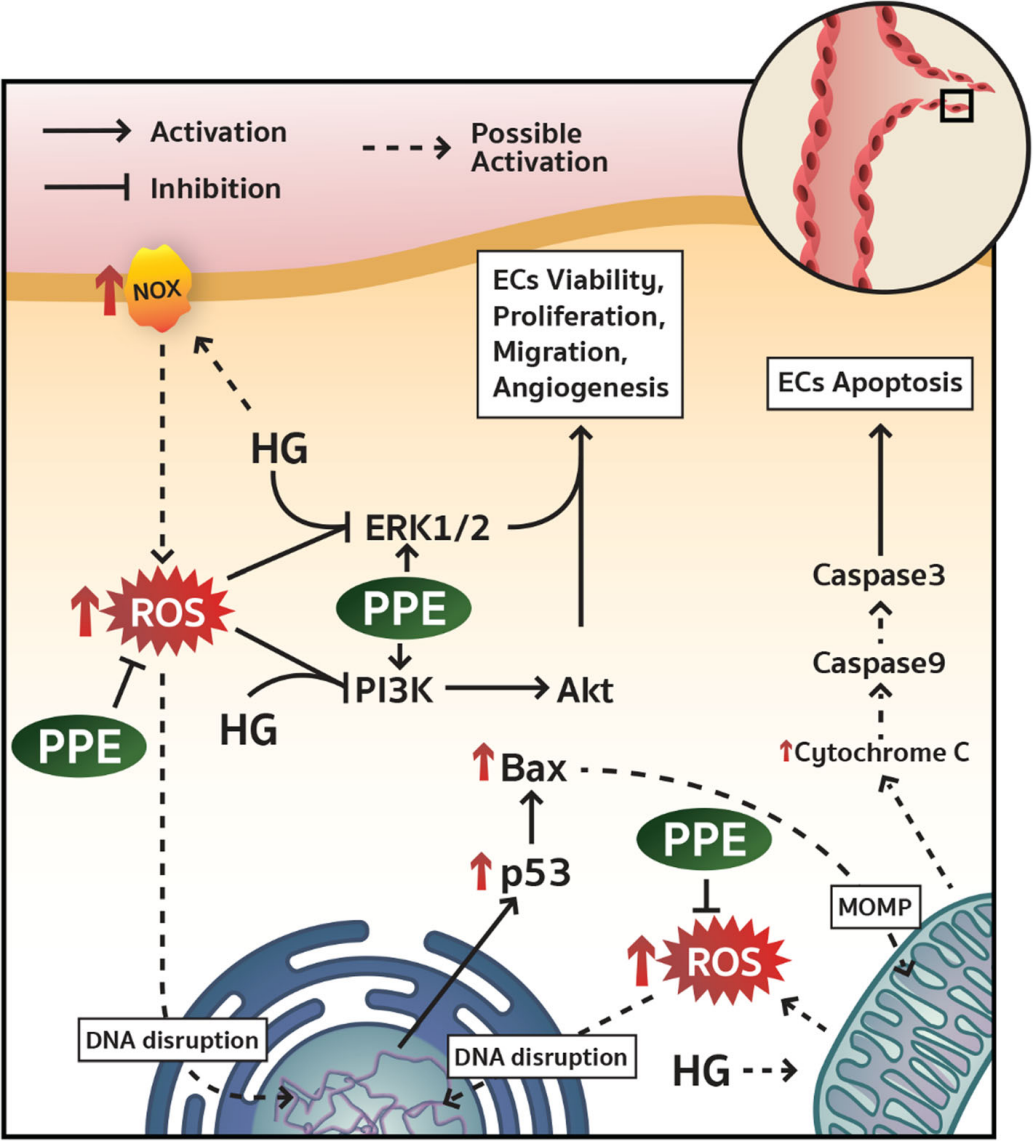
A schematic of stimulatory effect of PPE on HG-induced ROS-mediated angiogenesis impairment. PPE ameliorated HG-induced ROS-mediated ECs viable, migrative, and angiogenesis impairment by showing recovery of PI3K/Akt/ERK1/2 activation. Additionally, PPE also attenuated HG-induced ROS-mediated ECs apoptosis by showing p53/Bax/Caspase9/Caspase3 inhibition

HG-induced ROS overproduction has been reported as a cause of ECs dysfunction [16, 17]. HG-induced ECs viable, migrative, and angiogenesis impairment has also been remarked as a consequence of intracellular ROS overproduction [18, 19]. The angiogenesis impairment is a pivotal factor that leads to delayed wound healing in poorly controlled diabetic patients [6]. Remarkably, PPE has demonstrated as a wound healing accelerator and ROS inhibitor in previous studies [13, 14]. Consequently, PPE may improve angiogenesis impairment through intracellular ROS inhibition and ECs viable and migrative stimulation in HG. In this study, ECs were treated with PPE in HG to investigate the stimulatory effect of PPE on angiogenesis. HG alone proves the evidence of ECs viable, migrative, and angiogenesis impairment through the intracellular ROS increment. The major finding in this study is the inhibitory effect of PPE on HG-induced ROS overproduction that leads to the maintenance of ECs viability, migration, and angiogenesis by showing recovery of PI3K/Akt/ERK1/2 activation. Additionally, the inhibitory effect of PPE on HG-induced ROS overproduction also attenuated apoptotic regulatory molecules; p53, Bax, cleaved caspase 9, and cleaved caspase 3 (Fig. 8).

The intracellular ROS overproduction plays a role in HG-induced glucotoxicity. The excessive glucose influx increases intracellular ROS through depriving redox pathway-related molecules [20]. Moreover, the NADPH oxidase (NOX)-derived ROS in ECs has been implicated a major consequence of HG exposure. HG exposure activates NOX overexpression at the ECs membrane, whereby it leads to the superoxide generation along with endothelial nitric oxide synthase (eNOS) uncoupling. The eNOS uncoupling enhances reactive nitrogen species (RNS), which activates oxidative stress [21–23]. This excessive ROS directly disrupts DNA and induces GAPDH blockage through poly (ADP-ribose) polymerase (PARP)-induced free-nicotinamide adenine dinucleotide (NAD^+^) depletion [24]. Since ROS-induced GAPDH blockage inhibits glyceraldehyde 3-phosphate (G3P) conversion, the upstream glycolysis converts to alternative pathways that are defined as glucotoxicity; polyol pathway, glucosamine pathway, protein kinase C (PKC) pathway, and advanced-glycation end product (AGE) pathway [25–27]. Remarkably, the homeostasis of intracellular ROS is seemingly a key for improving HG-induced ECs dysfunction. The findings from current studies elucidate that the inhibition of HG-induced ROS overproduction preserves ECs viability, migration, angiogenesis and improves ECs injury [28–30].

Angiogenesis comprises many processes to form a new capillary. ECs are initially activated by pro-angiogenic signals, which convey the metalloprotease production along with vascular fenestration. Subsequently, ECs proliferate and migrate to form a tube and mature vessels. Angiogenesis impairment has been clarified as a consequence of HG-deprived ECs viability and migration, which is a consequence of HG-induced intracellular ROS overproduction [19, 31]. The findings from current studies showed angiogenic growth factors and hormones improve HG-induced ECs impairment [32–34]. Our PPE also characterized the protein component by LC MS/MS and searched data against the domestic porcine *Sus scofa* proteome database. Our PPE contains 391 protein sequences from 447 of total proteins. The analysis of DAVID bioinformatics resources elucidated that our PPE mostly comprised signal-involved proteins, phosphoproteins, and disulfide-bond proteins [35]. Accordingly, our PPE may consist of essential growth factors and antioxidative agents or reductant enzymes that provide bioactive functions.

The inhibition of the pro-survival signaling pathway has been illustrated as the molecular signaling of HG-induced ROS-mediated angiogenesis impairment in many studies [29, 36, 37]. The excessive ROS inhibits the PI3K/Akt signaling pathway, whereby it leads to insulin resistance and ECs survival reduction [38]. The ROS-induced Akt downregulation also implicated ECs migrative impairment [39]. In this study, HG reduced phosphorylation of PI3K and Akt compared to NG, whereas PPE increased phosphorylation of PI3K and Akt in HG. It suggests PPE ameliorated HG-reduced PI3K and Akt activation through intracellular ROS inhibition and direct enhancement of PI3K and Akt activation. This improvement of the PI3K and Akt signaling pathway eventually preserves ECs viability and migration in HG. Furthermore, HG also reduced phosphorylation of ERK1/2 compared to NG, whereas PPE increased phosphorylation of ERK1/2 in HG. It suggests PPE ameliorated HG-reduced ERK1/2 activation through intracellular ROS inhibition and direct enhancement of ERK1/2 activation. Moreover, the investigation of apoptotic regulatory molecules such as p53, Bax, Bcl 2, cleaved caspase 9, and cleaved caspase 3 was performed to elucidate the inhibitory effect of PPE on HG-induced ECs apoptosis. Many studies have indicated HG-induced ROS-mediated ECs apoptosis [40, 41]. In this study, HG increased p53, Bax, cleaved caspase 9, and cleaved caspase 3 levels compared to NG, whereas PPE reduced p53, Bax, cleaved caspase 9, and cleaved caspase 3 levels in HG. Particularly, PPE increased Bcl 2 level in HG and NAC also reduced p53, Bax, cleaved caspase 9, and cleaved caspase 3. It suggests that PPE potentially attenuated HG-induced ROS-mediated ECs apoptosis. The angiogenic growth factors secretion was also determined in this study. PPE ameliorated HG-reduced bFGF and EGF secretion, which are pivotal signals for angiogenesis activation [42]. Unfortunately, VEGF could not be detected in the supernatant. PPE may consist a few activators of VEGF gene transcription or may not directly affect pro-angiogenic growth factor secretion. However, the determination of intracellular protein and gene expression of pro-angiogenic growth factor may be considered in the future.

The limitation of this study is the using of an in vitro model for investigating HG-induced angiogenesis impairment. The in vivo model of angiogenesis may mimic to the organism, which presents a complete and fully functional angiogenic process. However, the determination of angiogenesis by using the in vitro tube formation assay provides reproducibility rather than other the in vivo model [43]. Remarkably, this is the first study that elucidates the stimulatory effect of PPE on HG-induced angiogenesis impairment and its molecular mechanisms. Since PPE greatly comprises the abundance of growth factors, nutrients, antioxidants, etc., the purification into one agent may debase its integrated bioactive functions. Consequently, the integrated PPE is emphasized rather than a single agent in our study. The investigation of PPE’s stimulatory effect on angiogenesis and wound healing in animal model may require prior to clinical application. Therefore, the future work should focus on angiogenesis and wound healing in a diabetic rat model. The wound biopsy should be stained with CD31 for angiogenesis evaluation in a wound area. The wound tissue should be also homogenized to determine the pro-survival signaling pathway and the apoptotic regulatory molecules.

Since angiogenesis impairment has been implicated as one pivotal factor of delayed wound healing in poorly controlled diabetic patients, whereby the risk of amputation is higher. The topical PPE treatment on the wound site may provide a better outcome of wound repair including preventive potential of amputation in diabetic patients.

## Conclusion

This is the first study that demonstrates the stimulatory effect of PPE on HG-induced angiogenesis impairment and its molecular mechanisms. The PPE attenuated HG-induced ROS overproduction and ameliorated ECs viability, migration, and angiogenesis by showing recovery of PI3K/Akt/ERK1/2 activation. Additionally, the inhibitory effect of PPE on HG-induced ROS overproduction also prevents ECs apoptosis.

## Supplementary Information

**Additional file 1.** Full-lenght-blotsR1

## Data Availability

The datasets used and/or analyzed during the current study are available from the corresponding author on reasonable requests.

## Abbreviations

PPE: Porcine placenta extract
HG: High glucose
NG: Normal glucose
NAC: N-acetylcysteine
ROS: reactive oxygen species
HUVEC: Human umbilical vein endothelial cell
ECs: Endothelial cells
H_2_O_2_: Hydrogen peroxide
MTT: 3-[4,5-dimethylthiazol-2-yl]-2,5-diphenyltetrazolium bromide
DCFDA: 2′,7′-Dichlorodi-hydrofluorescein diacetate
PVDF: Polyvinylidene fluoride
PKC: Protein kinase C
AGEs: Advanced glycation end products
NADPH: Nicotinamide adenine dinucleotide phosphate
LC MS/MS: Liquid Chromatography with tandem mass spectrometry
bFGF: Basic fibroblast growth factor
EGF: Epidermal growth factor
VEGF: Vascular endothelial growth factor
PI3K: Phosphoinositide 3-kinase
Akt: Protein kinase B
ERK1/2: Extracellular signal-regulated kinases
Bax: Bcl-2-associated X protein
Clv: Cleaved
MOMP: Mitochondria outer membrane permeability

## References

[1] Tandara AA, Mustoe TA (2004). Oxygen in wound healing—more than a nutrient. World J Surg.

[2] Li J, Zhang Y-P, Kirsner RS (2003). Angiogenesis in wound repair: Angiogenic growth factors and the extracellular matrix. Microsc Res Tech.

[3] Bauer SM, Bauer RJ, Velazquez OC (2005). Angiogenesis, Vasculogenesis, and induction of healing in chronic wounds. Vasc Endovasc Surg.

[4] Tahergorabi Z, Khazaei M (2012). Imbalance of angiogenesis in diabetic complications: the mechanisms. Int J Prev Med.

[5] Simo R, Carrasco E, Garcia-Ramirez M, Hernandez C (2006). Angiogenic and Antiangiogenic factors in proliferative diabetic retinopathy. Curr Diab Rev.

[6] Polverini PJ (2011). Angiogenesis and wound healing: basic discoveries, clinical implications, and therapeutic opportunities. Endod Top.

[7] Liu H, Yu S, Zhang H, Xu J (2012). Angiogenesis impairment in diabetes: role of Methylglyoxal-induced receptor for advanced Glycation Endproducts, autophagy and vascular endothelial growth factor receptor 2. PLoS One.

[8] Crawford TN, Alfaro Iii DV, Kerrison JB, Jablon EP (2009). Diabetic retinopathy and angiogenesis. Curr Diabetes Rev.

[9] Schieber M, Chandel NS (2014). ROS function in redox signaling and oxidative stress. Curr Biol.

[10] Fadini GP, Sartore S, Baesso I, Lenzi M, Agostini C, Tiengo A (2006). Endothelial progenitor cells and the diabetic paradox. Diabetes Care.

[11] Zhao Y, Singh RP. The role of anti-vascular endothelial growth factor (anti-VEGF) in the management of proliferative diabetic retinopathy. Drugs Context. 2018;7:–212532. 10.7573/dic.212532 PMC6113746 30181760

[12] Abdallah W, Fawzi AA (2009). Anti-VEGF therapy in proliferative diabetic retinopathy. Int Ophthalmol Clin.

[13] Wu C-H, Chang G-Y, Chang W-C, Hsu C-T, Chen R-S (2003). Wound healing effects of porcine placental extracts on rats with thermal injury. Br J Dermatol.

[14] Heo JH, Heo Y, Lee HJ, Kim M, Shin HY (2018). Topical anti-inflammatory and anti-oxidative effects of porcine placenta extracts on 2,4-dinitrochlorobenzene-induced contact dermatitis. BMC Complement Altern Med.

[15] Kongpol K, Nernpermpisooth N, Prompunt E, Kumphune S (2019). Endothelial-cell-derived human secretory leukocyte protease inhibitor (SLPI) protects Cardiomyocytes against ischemia/reperfusion injury. Biomolecules..

[16] Shenouda SM, Widlansky ME, Chen K, Xu G, Holbrook M, Tabit CE (2011). Altered mitochondrial dynamics contributes to endothelial dysfunction in diabetes mellitus. Circulation..

[17] Szewczyk A, Jarmuszkiewicz W, Koziel A, Sobieraj I, Nobik W, Lukasiak A (2015). Mitochondrial mechanisms of endothelial dysfunction. Pharmacol Rep.

[18] Zhu ZX, Cai WH, Wang T, Ye HB, Zhu YT, Chi LS (2015). bFGF-regulating MAPKs are involved in high glucose-mediated ROS production and delay of vascular endothelial cell migration. PLoS One.

[19] Rezabakhsh A, Ahmadi M, Khaksar M, Montaseri A, Malekinejad H, Rahbarghazi R (2017). Rapamycin inhibits oxidative/nitrosative stress and enhances angiogenesis in high glucose-treated human umbilical vein endothelial cells: role of autophagy. Biomed Pharmacother.

[20] Zhang Z, Yang Z, Zhu B, Hu J, Liew CW, Zhang Y (2012). Increasing glucose 6-phosphate dehydrogenase activity restores redox balance in vascular endothelial cells exposed to high glucose. PLoS One.

[21] Montezano AC, Touyz RM (2012). Reactive oxygen species and endothelial function – role of nitric oxide synthase uncoupling and Nox Family Nicotinamide adenine dinucleotide phosphate oxidases. Basic Clin Pharmacol Toxicol.

[22] Gao L, Mann GE (2009). Vascular NAD(P) H oxidase activation in diabetes: a double-edged sword in redox signalling. Cardiovasc Res.

[23] Ding H, Aljofan M, Triggle CR (2007). Oxidative stress and increased eNOS and NADPH oxidase expression in mouse microvessel endothelial cells. J Cell Physiol.

[24] Du X, Matsumura T, Edelstein D, Rossetti L, Zsengellér Z, Szabó C (2003). Inhibition of GAPDH activity by poly (ADP-ribose) polymerase activates three major pathways of hyperglycemic damage in endothelial cells. J Clin Invest.

[25] Ighodaro OM (2018). Molecular pathways associated with oxidative stress in diabetes mellitus. Biomed Pharmacother.

[26] Pricci F, Leto G, Amadio L, Iacobini C, Cordone S, Catalano S (2003). Oxidative stress in diabetes-induced endothelial dysfunction involvement of nitric oxide and protein kinase C. Free Radic Biol Med.

[27] Incalza MA, D'Oria R, Natalicchio A, Perrini S, Laviola L, Giorgino F (2018). Oxidative stress and reactive oxygen species in endothelial dysfunction associated with cardiovascular and metabolic diseases. Vasc Pharmacol.

[28] Li Q, Lin Y, Wang S, Zhang L, Guo L (2017). GLP-1 inhibits high-glucose-induced oxidative injury of vascular endothelial cells. Sci Rep.

[29] Duan M-X, Zhou H, Wu Q-Q, Liu C, Xiao Y, Deng W, et al. Andrographolide protects against HG-induced inflammation, apoptosis, migration, and impairment of angiogenesis via PI3K/AKT-eNOS Signalling in HUVECs. Mediat Inflamm. 2019:6168340. 10.1155/2019/6168340 PMC6800917 31686985

[30] Zhou D-Y, Su Y, Gao P, Yang Q-H, Wang Z, Xu Q (2015). Resveratrol ameliorates high glucose-induced oxidative stress injury in human umbilical vein endothelial cells by activating AMPK. Life Sci.

[31] Rezabakhsh A, Montazersaheb S, Nabat E, Hassanpour M, Montaseri A, Malekinejad H (2017). Effect of hydroxychloroquine on oxidative/nitrosative status and angiogenesis in endothelial cells under high glucose condition. BioImpacts.

[32] Han J, Mandal AK, Hiebert LM (2005). Endothelial cell injury by high glucose and heparanase is prevented by insulin, heparin and basic fibroblast growth factor. Cardiovasc Diabetol.

[33] Yang Z, Mo X, Gong Q, Pan Q, Yang X, Cai W (2008). Critical effect of VEGF in the process of endothelial cell apoptosis induced by high glucose. Apoptosis..

[34] Song H, Wu F, Zhang Y, Zhang Y, Wang F, Jiang M (2014). Irisin promotes human umbilical vein endothelial cell proliferation through the ERK signaling pathway and partly suppresses high glucose-induced apoptosis. PLoS One.

[35] Padhomchai Pumbthongthae PH, Sittiruk Roytrakul, Tavan Janvilisri, Puey Ounjai, editor Analysis of Protein Profile of Crude porcine placenta extract. The 50th National Graduate Research Conference; 7 2020; King Mongkut's Institute of Technology Ladkrabang, Bangkok.

[36] Xing Y, Lai J, Liu X, Zhang N, Ming J, Liu H (2017). Netrin-1 restores cell injury and impaired angiogenesis in vascular endothelial cells upon high glucose by PI3K/AKT-eNOS. J Mol Endocrinol.

[37] Maamoun H, Benameur T, Pintus G, Munusamy S, Agouni A (2019). Crosstalk Between Oxidative Stress and Endoplasmic Reticulum (ER) Stress in Endothelial Dysfunction and Aberrant Angiogenesis Associated With Diabetes: A Focus on the Protective Roles of Heme Oxygenase (HO)-1. Front Physiol.

[38] De Nigris V, Pujadas G, La Sala L, Testa R, Genovese S, Ceriello A (2015). Short-term high glucose exposure impairs insulin signaling in endothelial cells. Cardiovasc Diabetol.

[39] Lamalice L, Boeuf FL, Huot J (2007). Endothelial cell migration during angiogenesis. Circ Res.

[40] Wu N, Shen H, Liu H, Wang Y, Bai Y, Han P (2016). Acute blood glucose fluctuation enhances rat aorta endothelial cell apoptosis, oxidative stress and pro-inflammatory cytokine expression in vivo. Cardiovasc Diabetol.

[41] Hou Q, Lei M, Hu K, Wang M (2015). The effects of high glucose levels on reactive oxygen species-induced apoptosis and involved signaling in human vascular endothelial cells. Cardiovasc Toxicol.

[42] Ucuzian AA, Gassman AA, East AT, Greisler HP (2010). Molecular mediators of angiogenesis. J Burn Care Res.

[43] Xie D, Ju D, Speyer C, Gorski D, Kosir MA (2016). Strategic endothelial cell tube formation assay: comparing extracellular matrix and growth factor reduced extracellular matrix. J Vis Exp.

