# Supplementary material for: Porcine placenta extract improves high-glucose-induced angiogenesis impairment
Source: BMC Complement Med Ther. 2021 Feb 18;21:66. doi: 10.1186/s12906-021-03243-z (PMC7893890; doi:10.1186/s12906-021-03243-z)
Supplement: Supplementary file 1 — Additional file 1. Full-lenght-blotsR1 [file 12906_2021_3243_MOESM1_ESM.pdf]

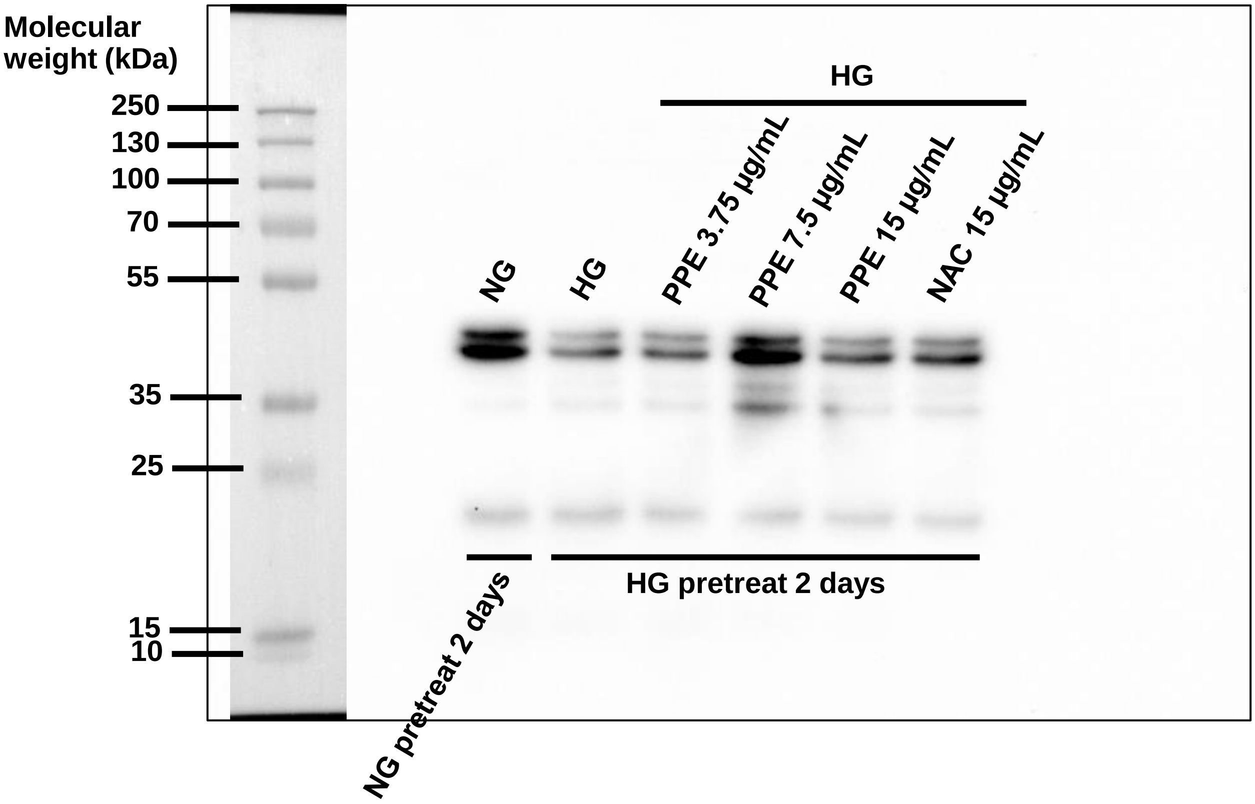

Supplementary Figure 1 The full-length blot of phosphorylated-ERK1/2 in HUVEC that is presented in figure 6

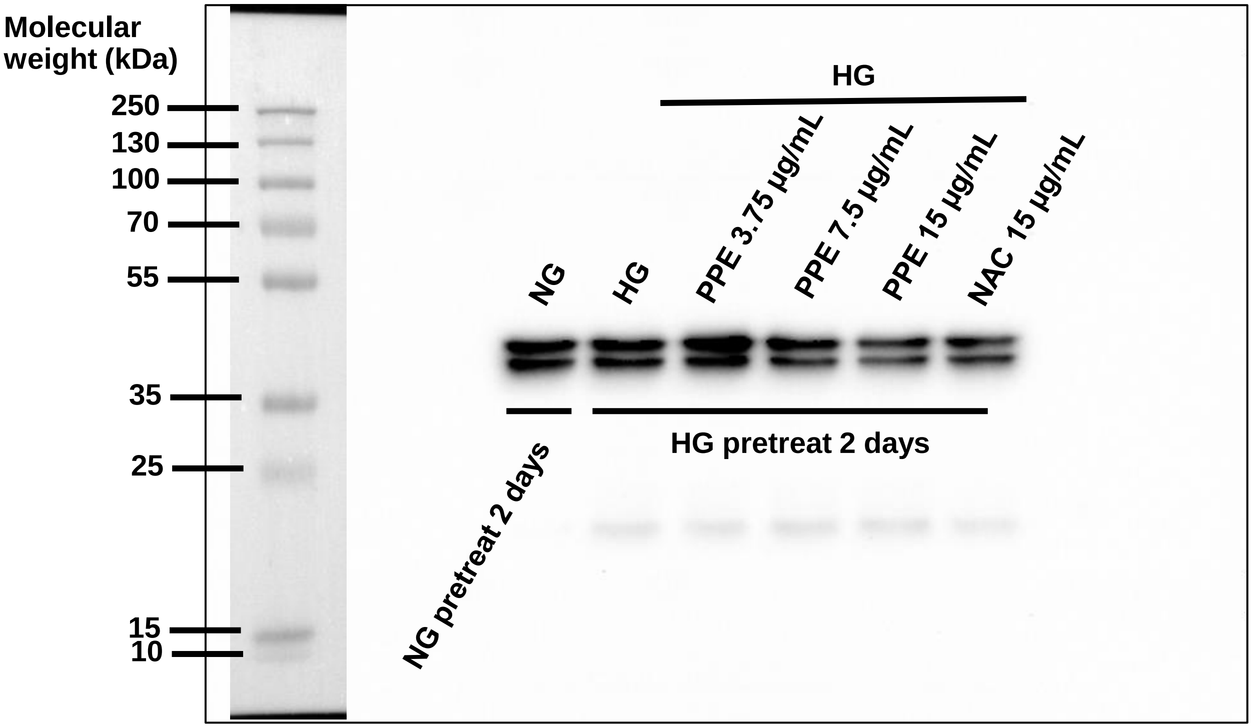

Supplementary Figure 2 The full-length blot of total ERK1/2 in HUVEC that is presented in figure 6

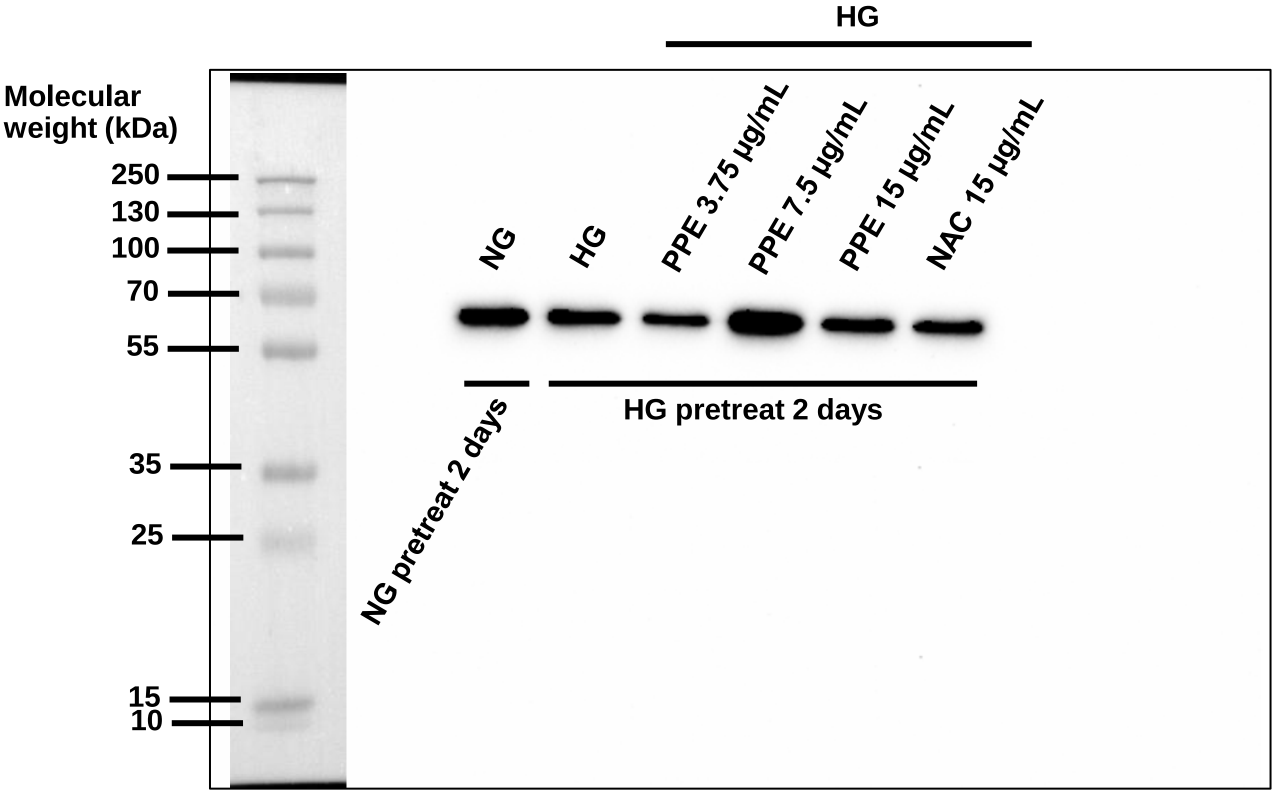

Supplementary Figure 3 The full-length blot of phosphorylated-Akt in HUVEC that is presented in figure 6

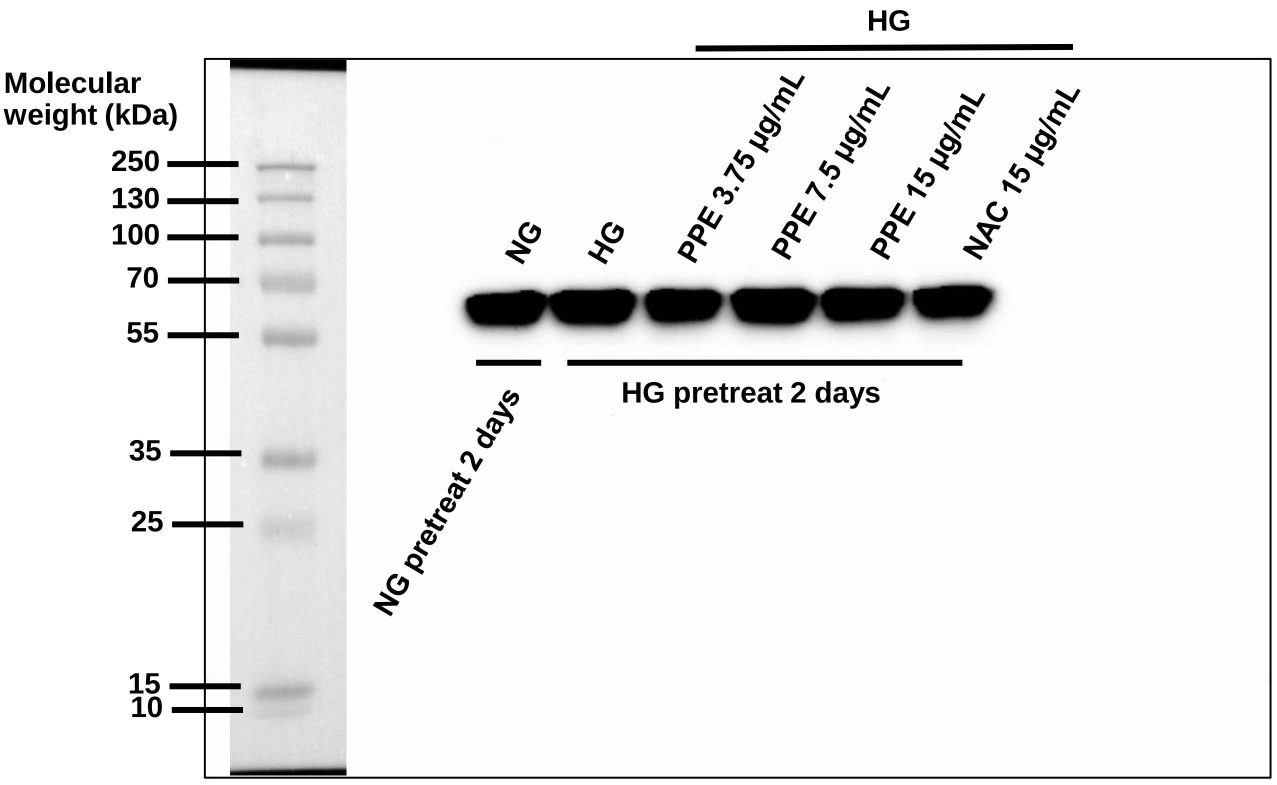

Supplementary Figure 4 The full-length blot of total Akt in HUVEC that is presented in figure 6

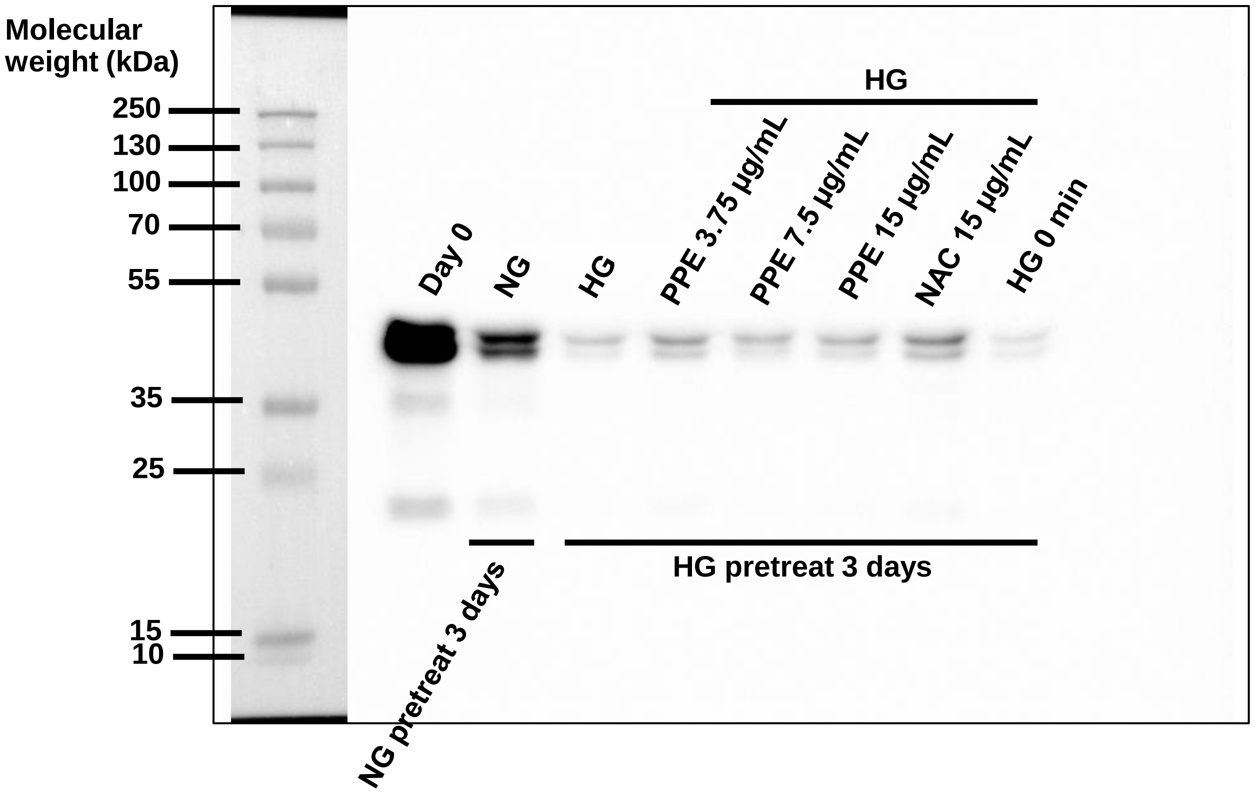

Supplementary Figure 5 The full-length blot of phosphorylated-ERK1/2 in EA.hy926 that is presented in figure 6

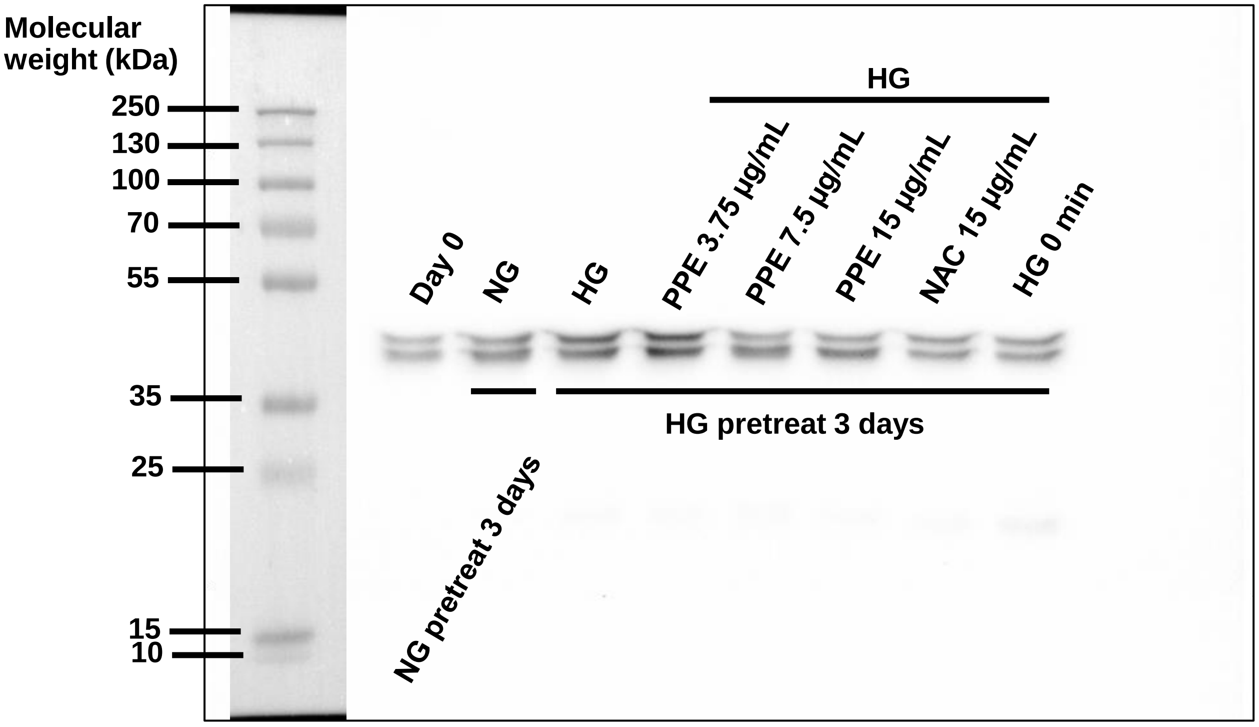

Supplementary Figure 6 The full-length blot of total ERK1/2 in EA.hy926 that is presented in figure 6

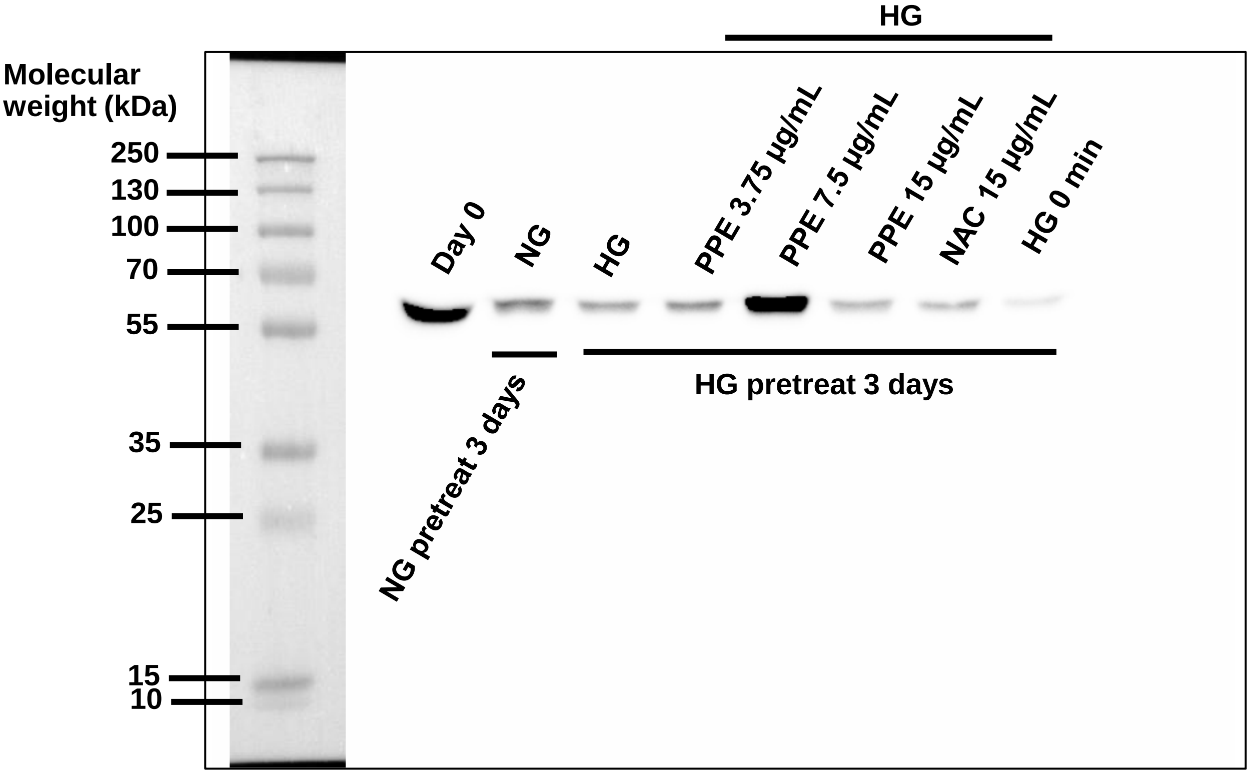

Supplementary Figure 7 The full-length blot of phosphorylated-Akt in EA.hy926 that is presented in figure 6

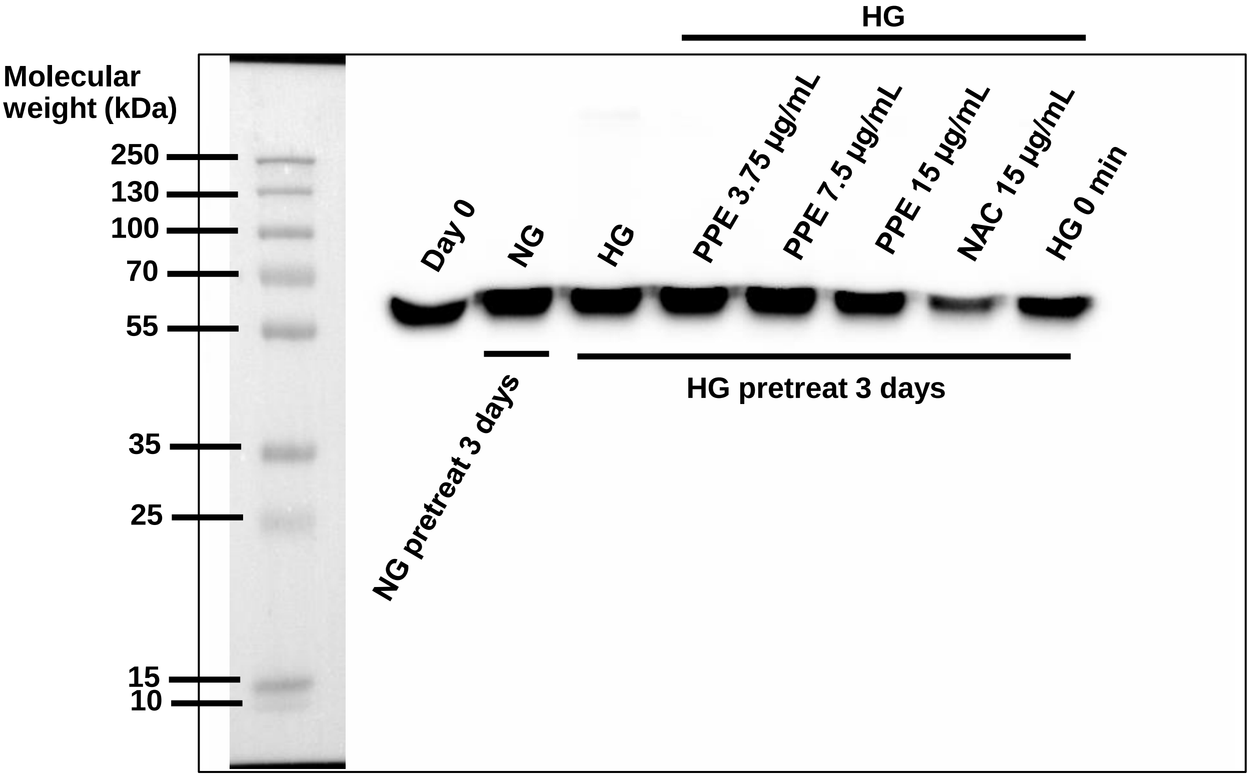

Supplementary Figure 8 The full-length blot of total Akt in EA.hy926 that is presented in figure 6

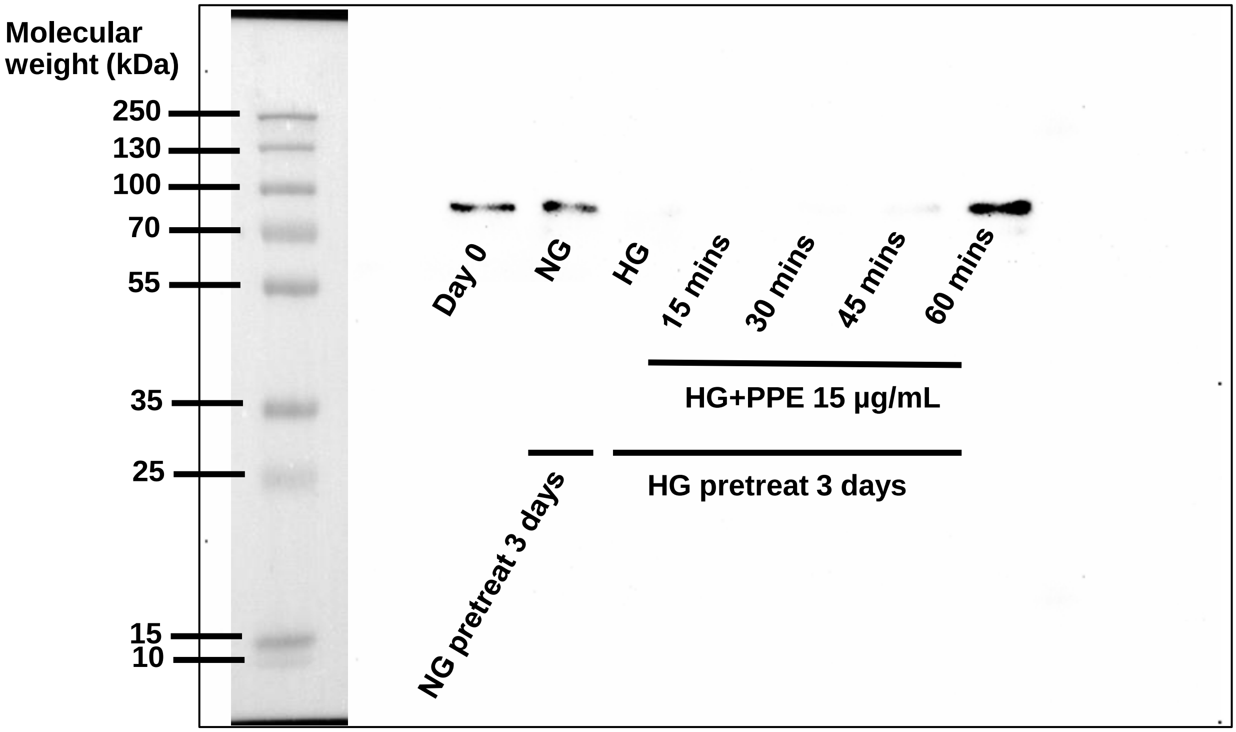

Supplementary Figure 9 The full-length blot of phosphorylated PI3K in EA.hy926 that is presented in figure 6

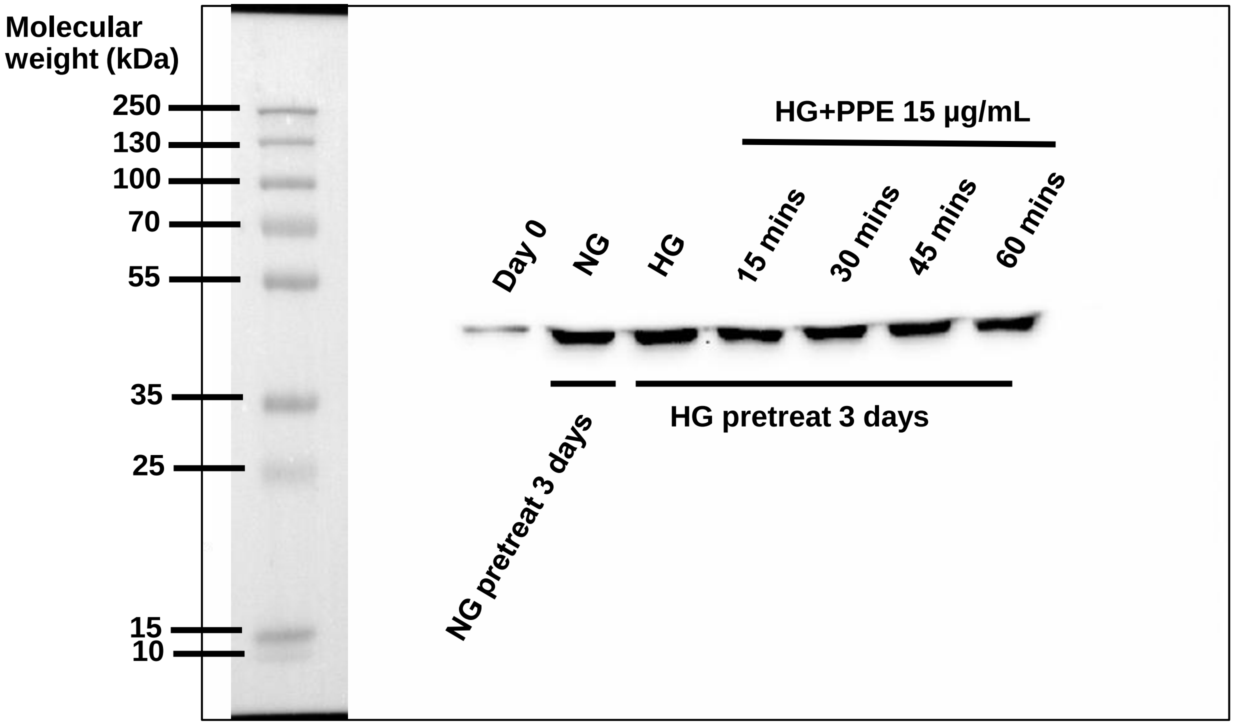

Supplementary Figure 10 The full-length blot of β-actin in EA.hy926 that is presented under cropped phospholyrated-PI3K blot in figure 6

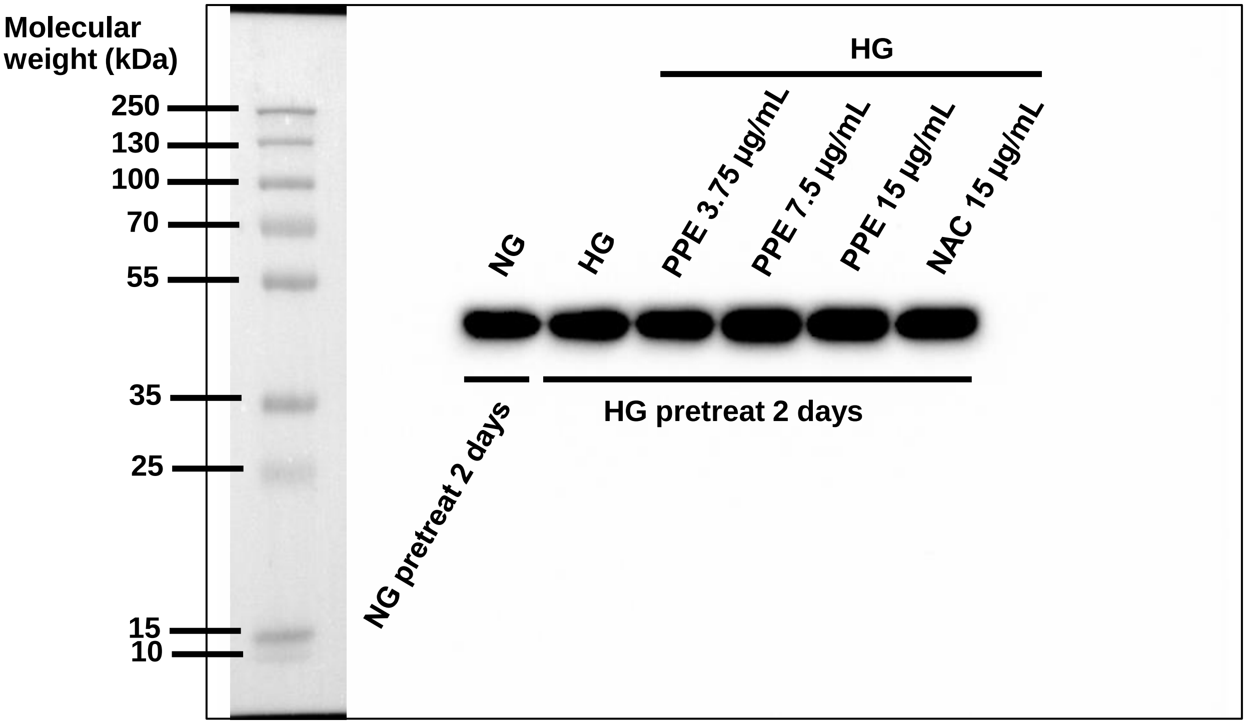

Supplementary Figure 11 The full-length blot of β-actin in HUVEC that is presented under cropped ERK1/2, Akt, phosphorylated-ERK1/2, phosphorylated-Akt blots in figure 6

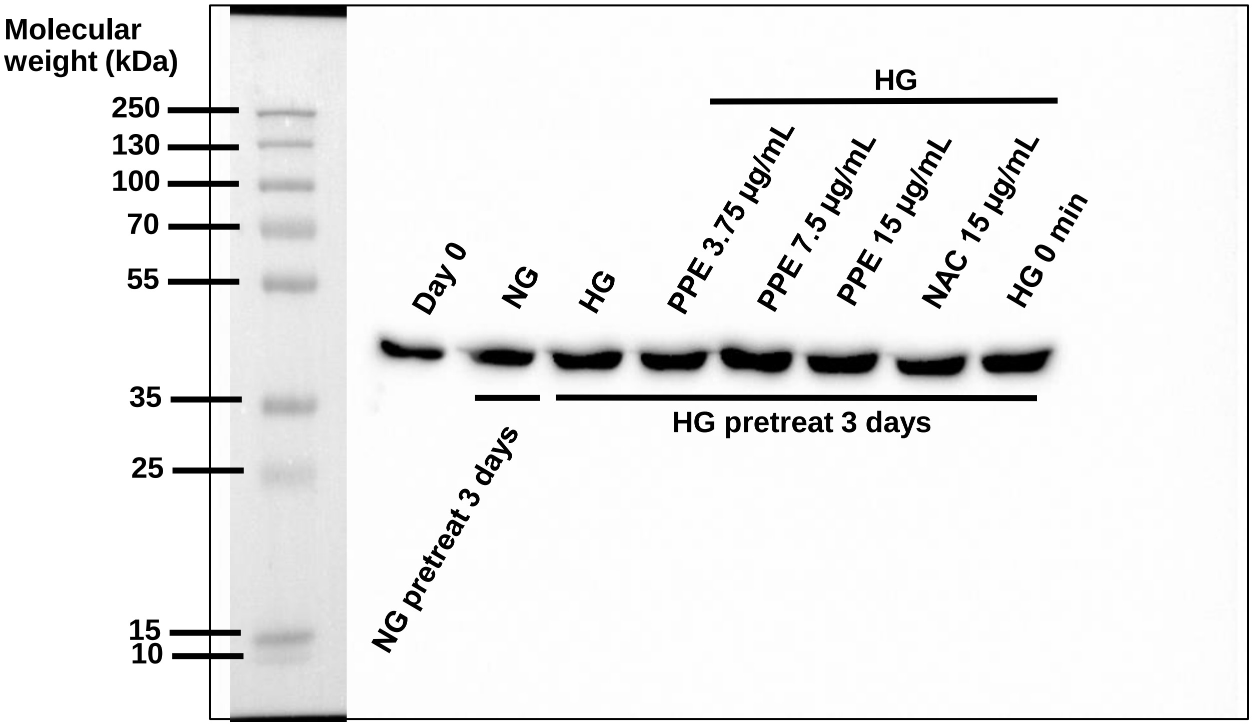

Supplementary Figure 12 The full-length blot of β-actin in EA.hy926 that is presented under cropped ERK1/2, Akt, phosphorylated-ERK1/2, phosphorylated-Akt blots in figure 6

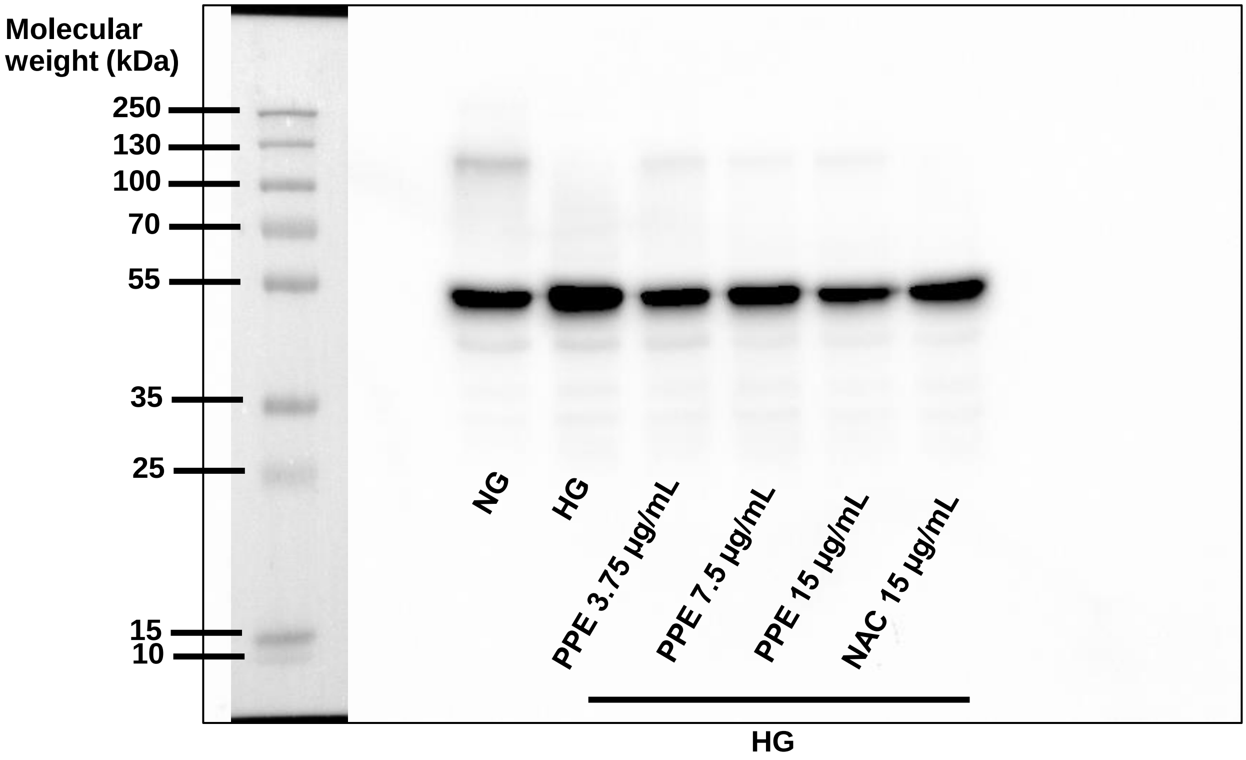

Supplementary Figure 13 The full-length blot of p53 that is presented in figure 7

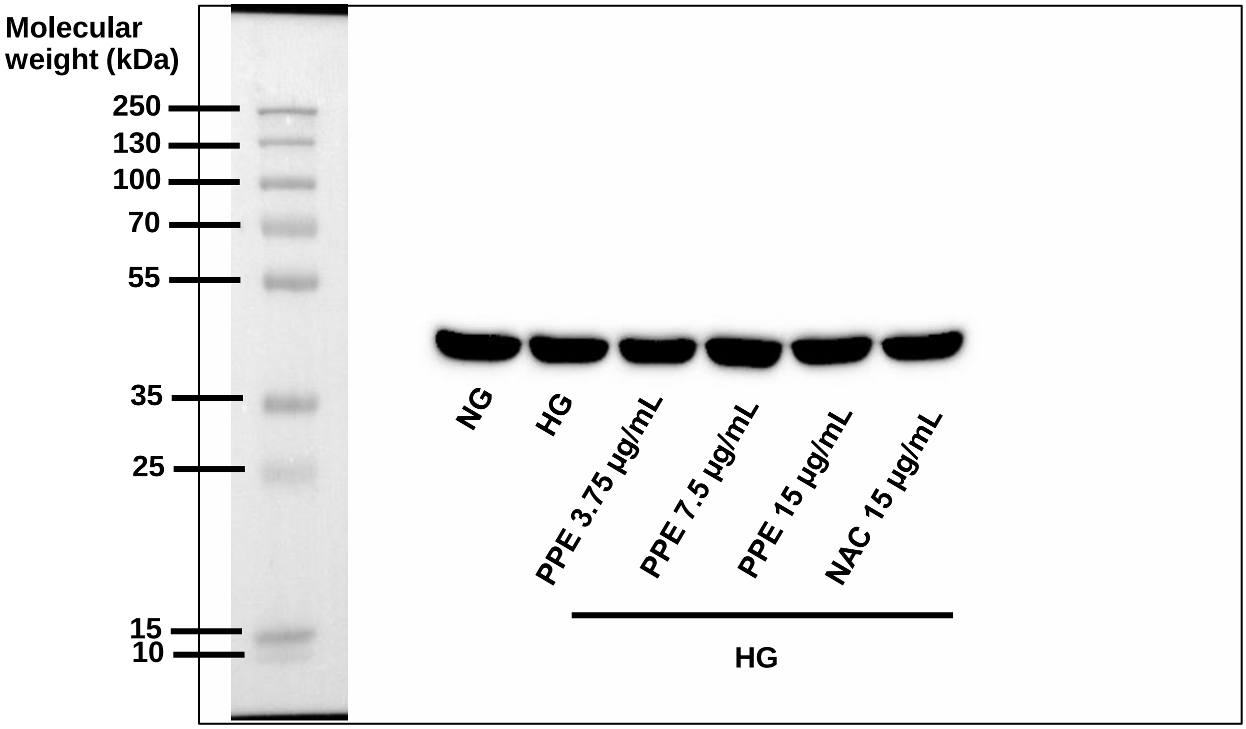

Supplementary Figure 14 The full-length blot of β-actin that is presented under cropped p53 blot in figure 7

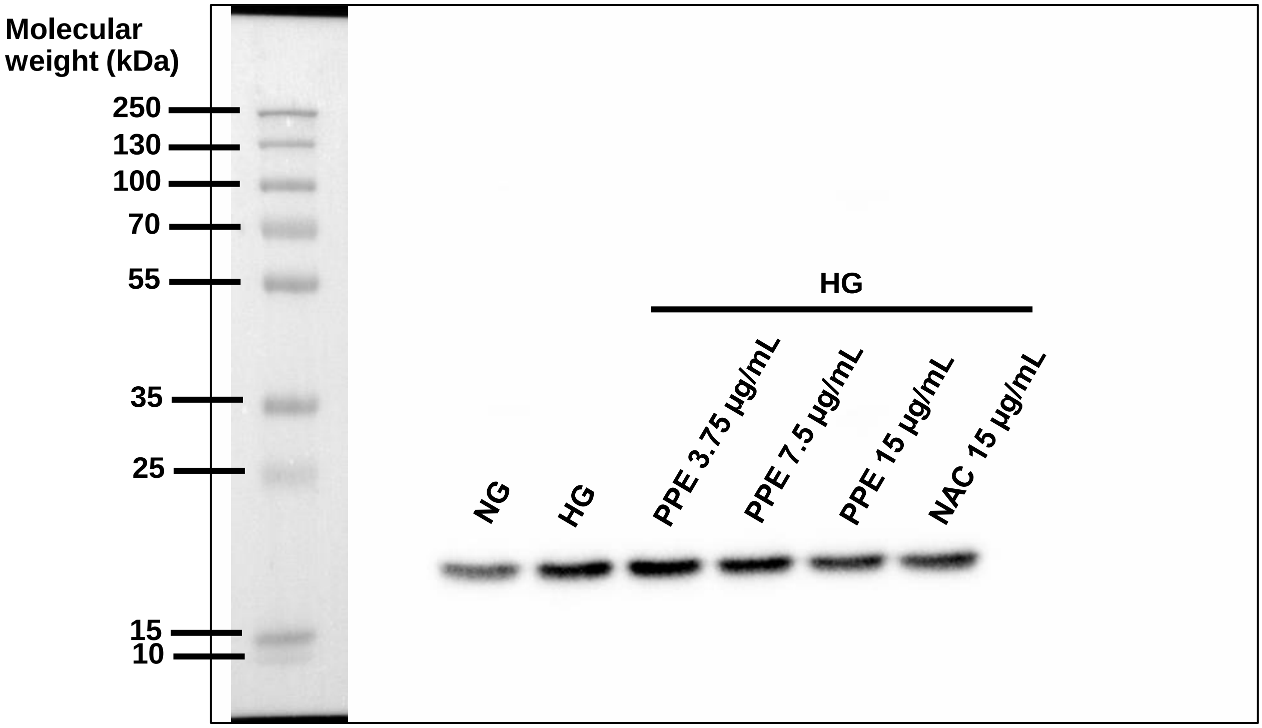

Supplementary Figure 15 The full-length blot of Bax that is presented in figure 7

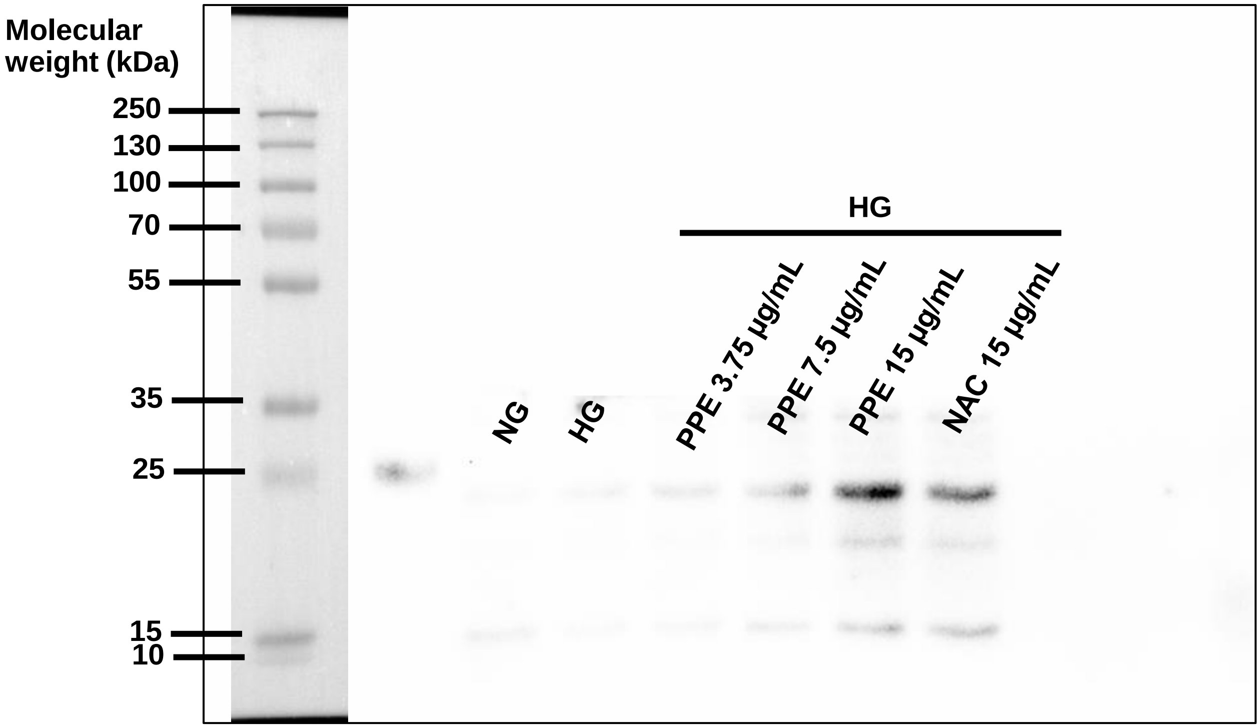

Supplementary Figure 16 The full-length blot of Bcl 2 that is presented in figure 7

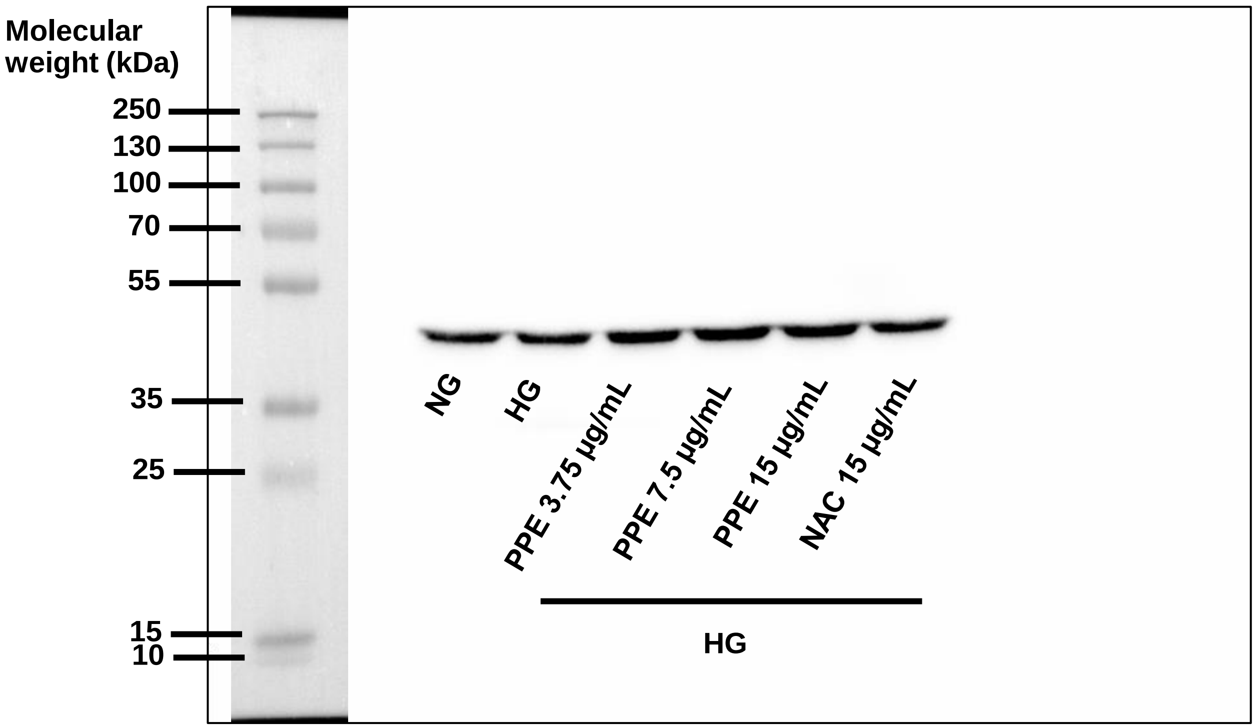

Supplementary Figure 17 The full-length blot of β-actin that is presented under cropped Bax and Bcl 2 blots in figure 7

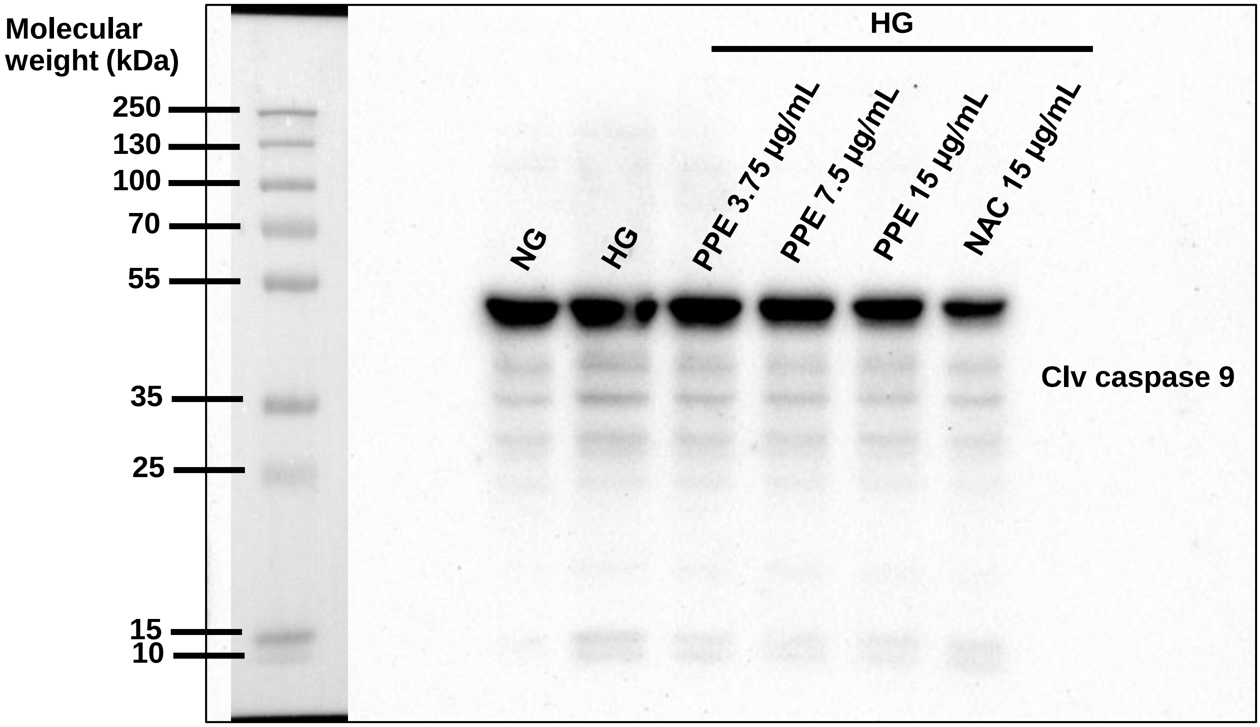

Supplementary Figure 18 The full-length blot of caspase 9 that is presented in figure 7

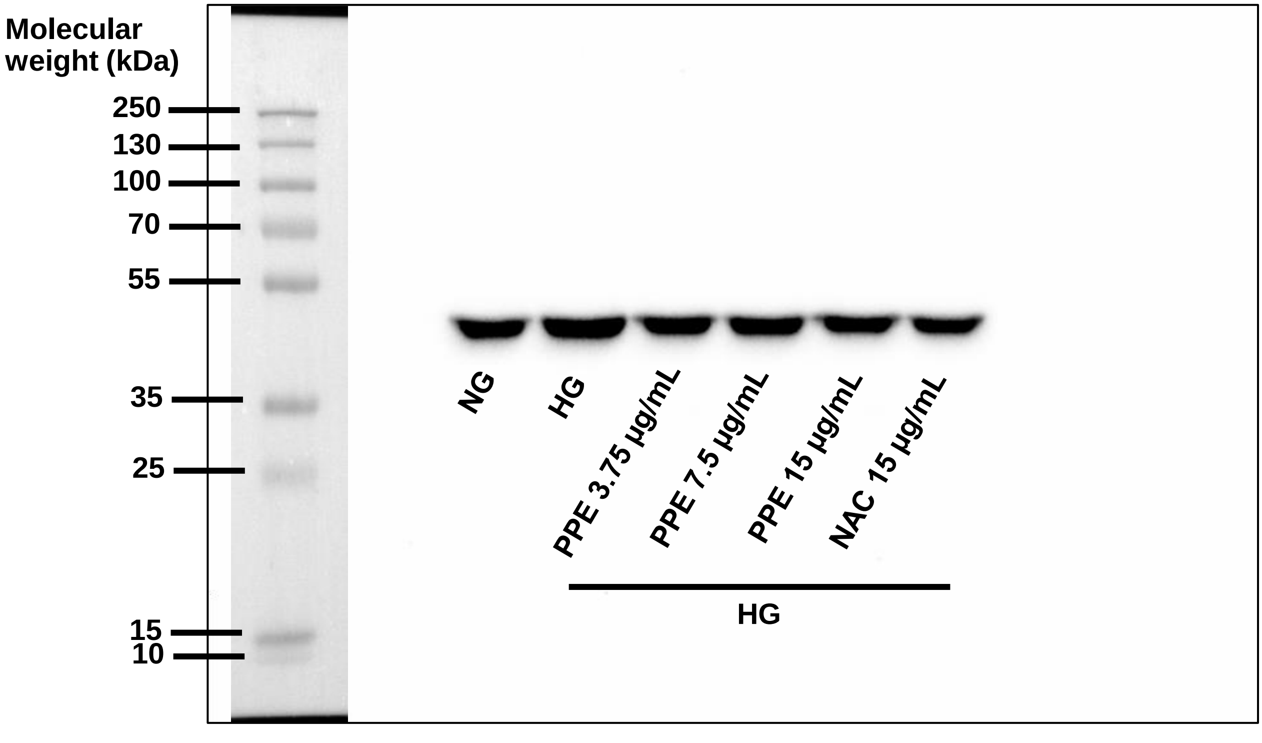

Supplementary Figure 19 The full-length blot of β-actin that is presented under cropped cleaved caspase 9 blot in figure 7

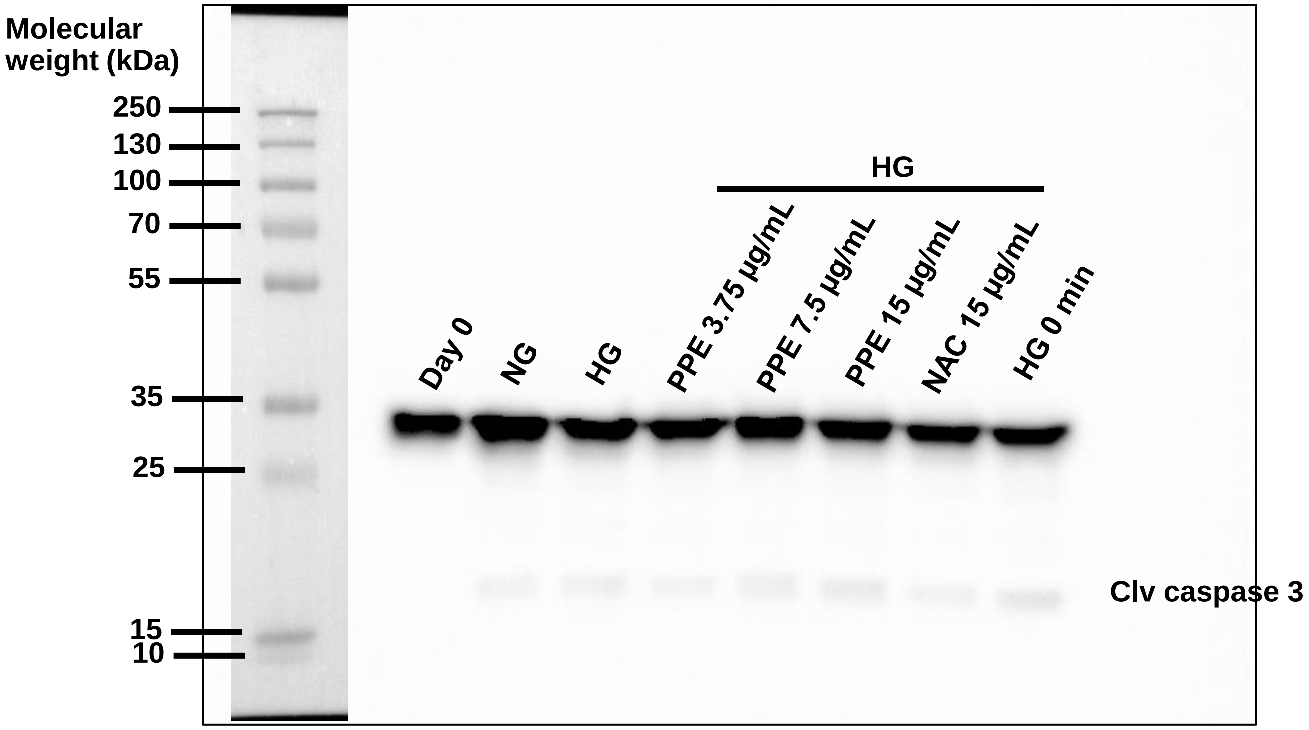

Supplementary Figure 20 The full-length blot of caspase 3 that is presented in figure 7

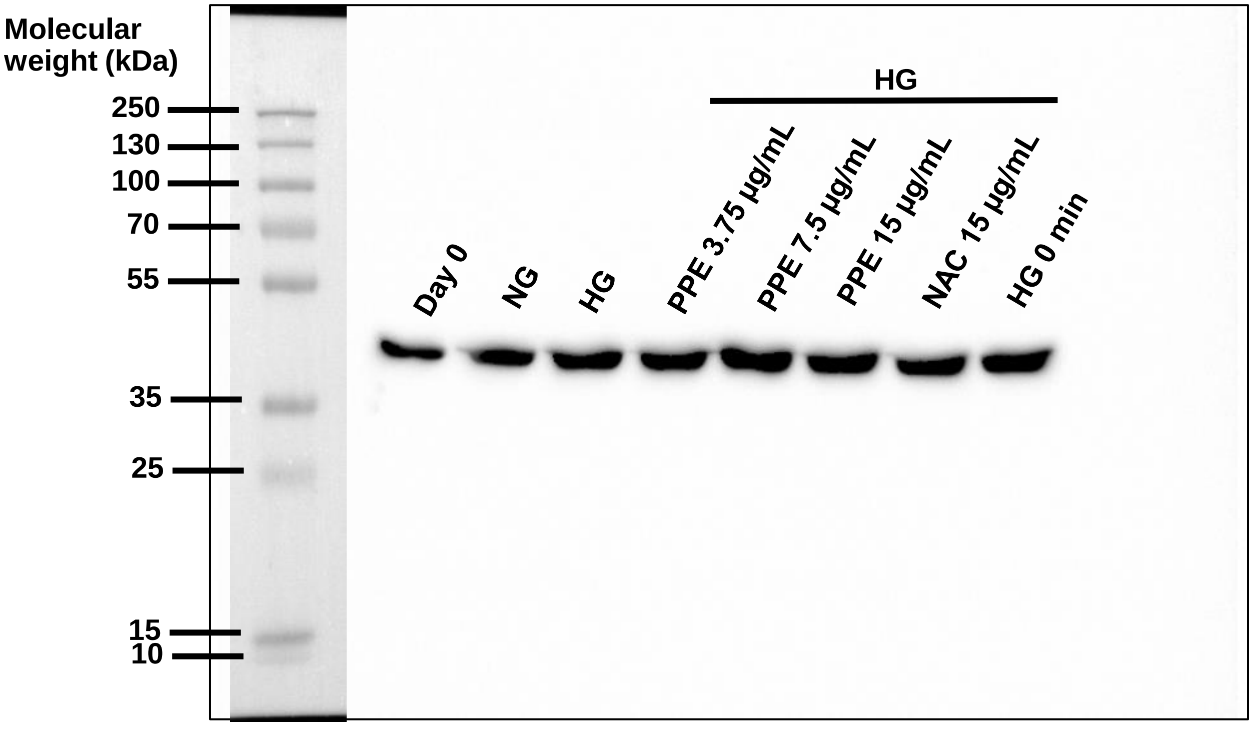

Supplementary Figure 21 The full-length blot of β-actin that is presented under cropped cleaved caspase 3 in figure 7
